# The mTOR Signalling Pathway in Human Cancer

**DOI:** 10.3390/ijms13021886

**Published:** 2012-02-10

**Authors:** Helena Pópulo, José Manuel Lopes, Paula Soares

**Affiliations:** 1 Institute of Molecular Pathology and Immunology of University of Porto (IPATIMUP), University of Porto, 4200-465, Porto, Portugal; E-Mails: hpopulo@ipatimup.pt (H.P.); jmlopes@ipatimup.pt (J.M.L.); 2 Medical Faculty, University of Porto, 4200-465 Porto, Portugal; 3 Department of Pathology, Hospital São João, 4200-465 Porto, Portugal

**Keywords:** mTOR, cancer, melanoma, therapy, rapamycin

## Abstract

The conserved serine/threonine kinase mTOR (the mammalian target of rapamycin), a downstream effector of the PI3K/AKT pathway, forms two distinct multiprotein complexes: mTORC1 and mTORC2. mTORC1 is sensitive to rapamycin, activates S6K1 and 4EBP1, which are involved in mRNA translation. It is activated by diverse stimuli, such as growth factors, nutrients, energy and stress signals, and essential signalling pathways, such as PI3K, MAPK and AMPK, in order to control cell growth, proliferation and survival. mTORC2 is considered resistant to rapamycin and is generally insensitive to nutrients and energy signals. It activates PKC-α and AKT and regulates the actin cytoskeleton. Deregulation of multiple elements of the mTOR pathway (*PI3K* amplification/mutation, *PTEN* loss of function, AKT overexpression, and S6K1, 4EBP1 and eIF4E overexpression) has been reported in many types of cancers, particularly in melanoma, where alterations in major components of the mTOR pathway were reported to have significant effects on tumour progression. Therefore, mTOR is an appealing therapeutic target and mTOR inhibitors, including the rapamycin analogues deforolimus, everolimus and temsirolimus, are submitted to clinical trials for treating multiple cancers, alone or in combination with inhibitors of other pathways. Importantly, temsirolimus and everolimus were recently approved by the FDA for the treatment of renal cell carcinoma, PNET and giant cell astrocytoma. Small molecules that inhibit mTOR kinase activity and dual PI3K-mTOR inhibitors are also being developed. In this review, we aim to survey relevant research, the molecular mechanisms of signalling, including upstream activation and downstream effectors, and the role of mTOR in cancer, mainly in melanoma.

## 1. Introduction

Cell behaviour is modulated by local circumstances: metabolism is hindered by the lack of nutrients and growth factors, with associated alterations in the expression of various genes involved in cellular physiology. Protein synthesis is consequently downregulated, thereby having a negative impact on growth and proliferation. An understanding of the mechanisms by which cells receive and integrate extracellular signals, triggering a cascade of intracellular signals that influence cell growth and metabolism, is essential to developing a well-targeted chemotherapy. One of these mechanisms is the mTOR signalling pathway, which links growth factors, nutrients and energy availability to cell survival, growth, proliferation, and motility (reviewed in refs. [1–3]).

The target of rapamycin (TOR) was originally discovered in the budding yeast *Saccharomyces cerevisiae*, as a target of the macrolide fungicide rapamycin, through mutants that showed growth resistance to rapamycin [4]. The structurally and functionally conserved mammalian counterpart (mTOR) was subsequently discovered biochemically based on its rapamycin inhibitory properties [5–7]. To date, every eukaryote genome examined (including yeasts, algae, plants, worms, flies and mammals) contains a TOR gene.

**Figure 1:**
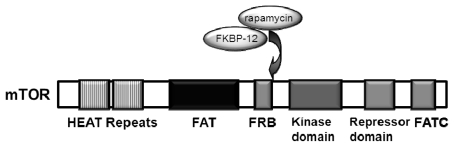
Domain structure of mTOR. The *N*-terminus of mTOR contains two tandem repeated HEAT motifs (protein interaction domains found in Huntington, Elongation factor 3, PR65/A and TOR), followed by a FAT (domain shared by FRAP, Ataxia telangiectasia mutated, and TRRAP, all of which are PIKK family members) domain, a FRB (FKBP12-rapamycin-binding site, found in all eukaryotic TOR orthologs) domain, a PtdIns 3-kinase related catalytic domain, an auto-inhibitory (repressor domain or RD domain), and a FATC (FAT *C* terminus) domain that is located at the *C*-terminus of the protein. The FRB domain forms a deep hydrophobic cleft that serves as the high-affinity binding site for the inhibitory complex FKBP12-rapamycin (Adapted from [10]).

mTOR (the mammalian target of rapamycin), also known as FRAP (FKBP12-rapamcyin-associated protein), RAFT1 (rapamycin and FKBP12 target), RAPT 1 (rapamycin target 1), or SEP (sirolimus effector protein), is a 289 kDa serine/threonine kinase [7] that belongs to the PI3K-related protein kinase (PIKKs) family, since its *C*-terminus shares strong homology to the catalytic domain of PI3K (Figure 1) [8,9].

**Figure 2:**
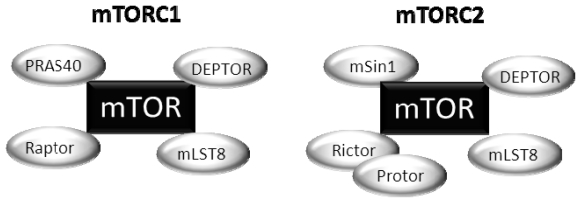
Schematic components of the mTOR complexes (mTORC). mTOR functions in two structural and functional distinct protein complexes: mTORC1, which contains two positive regulatory subunits, raptor and mLST8, and two negative regulators, PRAS40 and DEPTOR; and mTORC2, which contains rictor, mSin1 and Protor, and also mLST8 and DEPTOR.

mTOR encompasses two functionally distinct protein complexes: mTOR complex 1 and mTOR complex 2 (Figure 2) [11,12]. The mTORC1 consists of mTOR, raptor, mLST8, and two negative regulators, PRAS40 and DEPTOR [13–16]. Raptor regulates mTOR activity and functions as a scaffold for recruiting mTORC1 substrates [13,14]. Recent studies suggested that mTORC1 activity can be regulated by the phosphorylation status of raptor [17]. mLST8, another subunit of mTORC1, thought to bind to the kinase domain of mTOR, and to regulate positively the mTOR kinase activity, seems essential to maintain a nutrient and rapamycin-sensitive interaction between raptor and mTOR [18]. Other studies indicate that mLST8 is necessary for maintaining the rictor-mTOR interaction also in mTORC2 complex [19], leading to the proposal that mLST8 might be important for shuttling mTOR between the two mTOR complexes and consistent with the dynamic equilibrium of these complexes in mammalian cells [20]. PRAS40, another subunit of mTORC1, associates with mTORC1 via raptor and inhibits its activity [15]. A recent study identified DEPTOR as a mTOR-interacting protein [16]. DEPTOR interacts with both mTORC1 and mTORC2, negatively regulating their activities.

**Figure 3:**
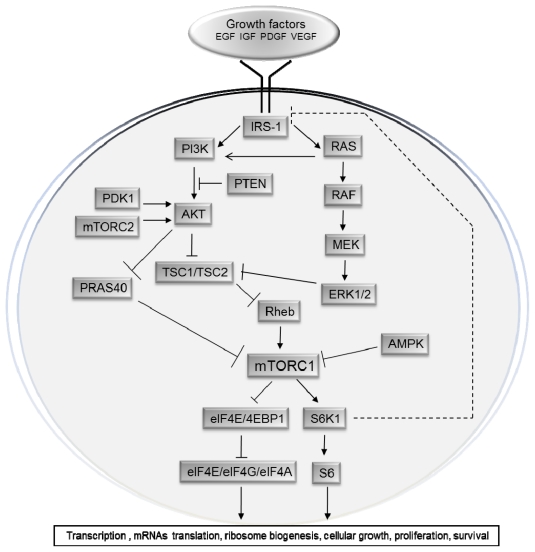
Diagram of the mTOR signalling pathway (see text for details). mTOR is a central regulator of cell growth and proliferation in response to environmental and nutritional conditions. The mTOR signalling pathway is regulated by growth factors, amino acids, and ATP and O_2_ levels. Signalling through mTOR modulates several downstream pathways that regulate cell-cycle progression, translation initiation, transcriptional stress responses, protein stability, and survival of cells.

mTORC1 is activated by the PI3K/AKT pathway (Figure 3) and inhibited by the TSC1/TSC2 complex; it is a major regulator of ribosomal biogenesis and protein synthesis [21], through the phosphorylation and activation of S6K, and the phosphorylation and inactivation of the repressor of mRNA translation 4EBP1. Since they are the best characterized downstream targets of mTOR, the phosphorylation status of S6K and 4EBP1 are commonly used to evaluate mTORC1 activity *in vivo*. In addition, mTORC1 is also involved in the regulation of other proteins including CLIP-170 (cytoplasm linker protein-170) [22], eEF2 (eukaryotic elongation factor 2) kinase [23], ODC (ornithine decarboxylase) [24], glycogen synthase [25], HIF-1 α (hypoxia-inducible factor 1α) [26], lipin [27], PKCδ and PKCɛ [28], PP2A (protein phosphatase 2A) [29], p21Cip1 and p27Kip1 cyclin-dependent kinase inhibitors [30,31], Rb (retinoblastoma) protein [32], and STAT3 (signal transducer and activator of transcription 3) [33].

mTORC2 contains mTOR, rictor, mLST8, mSin1, and the newly identified components Protor, Hsp70 and DEPTOR [12,34–36]. Rictor is an mTOR-associated protein that is exclusive from mTORC2 [12]. mLST8 is a stable component of both mTOR complexes [19]. mSin1 is an essential subunit of mTORC2, important for mTORC2 integrity and mTOR activity toward AKT Ser473 phosphorylation [35]. Protor-1 (protein observed with rictor-1) interacts with rictor, although it is not essential for the assembly of other mTORC2 subunits into the complex [36]. Hsp70, a heat shock protein, is required for the proper formation and kinase activity of mTORC2 under basal conditions and following heat shock [37]. DEPTOR is a negative regulator of both mTORC1 and mTORC2 [16].

mTORC2 is activated by growth factors, phosphorylates PKC-α, AKT (on Ser473) and paxillin (focal adhesion-associated adaptor protein), and regulates the activity of the small GTPases Rac and Rho related to cell survival, migration and regulation of the actin cytoskeleton [12,38,39]. Hence, mTORC2 and mTORC1 have different physiological functions.

The complexes differ in their sensitivity to the macrolide fungicide rapamycin; mTORC1 is sensitive and mTORC2 is deemed resistant. However, it was described that long-term treatment (over 24 h) with rapamycin can disrupt mTORC2 assembly and function by sequestering newly synthesized mTOR molecules [39].

The mTORC1 signalling cascade is activated by phosphorylated AKT (Figure 3). Class I PI3K produces the second messenger PtdIns(3,4,5)P3 [40]. PtdIns(3,4,5) P3 binds to the pleckstrin-homology (PH) domain of target proteins, including AKT and PDK1 [2]. Binding of PtdIns(3,4,5)P3 to the PH domain of AKT engages this kinase to the cell membrane where it is activated by phosphorylation at Thr308 by PDK1 [41,42], and by phosphorylation at Ser473 by mTORC2 [43], being both phosphorylation required for the full activation of AKT kinase activity [41]. PTEN is a negative regulator of AKT activation, as it converts PtdIns(3,4,5)P3 into PtdIns(4,5)P2, leading to a reduced recruitment of AKT to the cell membrane [44]. Activated AKT has several downstream substrates, including GSK3, FOXO transcription factors and TSC2 [45]. The phosphorylation of TSC2 prevents TSC1/TSC2 complex formation, which drives the small GTPase Rheb into the GTP-bound active state [40], leading to the activation of mTORC1 at Ser2448 [46,47]. The exact mechanism by which Rheb activates mTORC1 is unknown, but it was described to entail the interaction of GTP-bound Rheb with the amino-terminal lobe of the mTOR kinase domain [47] and the farnesylation and subsequent localization of Rheb in the Golgi and endomembranes [48,49]. AKT also phosphorylates and inhibits PRAS40, which negatively regulates mTORC1 by antagonizing its activation by Rheb [15,50].

Activated mTORC1 phosphorylates downstream effectors, including S6K1 and 4EBP1, via an interaction between raptor and a TOR signalling (TOS) motif in S6K and 4EBP [51–53]. The TOS motif is a conserved five amino acid segment found in the *N* terminus of S6K1 (Phe-Asp-Ile-Asp-Leu) and in the *C* terminus of 4E-BP1 (Phe-Glu-Met-Asp-Ile) that is necessary for the *in vivo* phosphorylation of these proteins by mTORC1 [54].

The serine/threonine kinase p70S6K1 is one of the most well-known downstream targets of mTORC1. S6K1 can also be activated by TOR-insensitive signalling pathways such as PDK1, MAPK and SAPK (stress-activated protein kinase). In spite of this, the phosphorylation of S6K1 at Thr389 by mTORC1 is required for its activation and the three phosphorylation sites identified of S6K1 can all be blocked by mTOR inhibitors [55]. Activated mTORC1 phosphorylates S6K1, which phosphorylates S6 (40S ribosomal protein S6), enhancing the translation of mRNAs with a 5′-terminal oligopolypyrimidine (5′-TOP). The targets of S6K1 include ribosomal proteins, elongation factors, and insulin growth factor 2 [56].

4EBP1 is another well-characterized mTORC1 target. 4EBP1 inhibits the initiation of protein translation by binding and inactivating eIF4E (eukaryotic translation initiation factor 4E) [57]. mTORC1 phosphorylates 4EBP1 at multiple sites to promote the dissociation of eIF4E from 4EBP1, relieving the inhibitory effect of 4EBP1 on eIF4E-dependent translation initiation [58]. Free eIF4E can form the multisubunit eIF4F complex binding to eIF4G (a large scaffolding protein), eIF4A (an ATP-dependent RNA helicase), and eIF4B, enabling cap-dependent protein translation, and inducing increased translation of mRNAs with regulatory elements in the 5′-untranslated terminal regions (5′-UTR) of its downstream target genes (e.g., c-myc, ornithine decarboxylase and cyclin D1), which are required for G1-to-S phase transition [56]. Differently, in quiescent cells or under low growth factors levels, unphosphorylated 4EBP1 binds to eIF4E, inhibiting the initiation of protein translation. The inhibition of mTOR by rapamycin also causes 4EBP1 dephosphorylation, which prevents protein translation [59].

Several studies suggest the existence of a negative feedback loop from the mTOR-S6K1 pathway to the upstream IRS pathway (Figure 3) [60,61]. Activation of mTORC1 and S6K1 regulates IRS-1 both at the transcriptional level and through direct phosphorylation on specific residues which prevent its recruitment and binding to RTKs, leading to a negative feedback regulation of both PI3K [62] and MAPK signalling [63].

In summary, the serine/threonine kinase mTOR, a downstream effector of the PI3K/AKT pathway, forms two complexes: mTORC1 and mTORC2. The complexes are constituted by different proteins and play distinct functions for cell maintenance. mTORC1 is sensitive to rapamycin, activates S6K1 and 4EBP1, which are involved in mRNA translation. mTORC2 is considered resistant to rapamycin, activates PKC-α and AKT and regulates the actin cytoskeleton.

## 2. Upstream Regulation of the mTOR Pathway

Growth factors and hormones, such as insulin, regulate mTORC1 signalling by the activation of class I PI3K and its downstream effector AKT, which reverses the inhibitory effect of TSC1/TSC2 complex and PRAS40 on mTORC1 signalling [64]. Stimulation of class I PI3K initiates several selective signalling cascades that lead to increased cell growth and proliferation [65].

mTORC1 can also be activated by nutrients. It was described that induction of S6K1 and 4EBP1 phosphorylation by amino acids depends on mTORC1 [66]. Another study described that amino-acid withdrawal results in the rapid dephosphorylation of S6K1 and 4EBP1, whereas addition of amino acids rescues this response in a rapamycin-sensitive manner [67]. Furthermore, it has been suggested that TSC1/TSC2 complex is not required for the regulation of mTORC1 by amino acids, although Rheb–GTP is required for this response [68]. Another mechanism by which amino acids may affect mTORC1 activity is via the class III PI3K, hVps34 (human vacuolar protein sorting-34). hVps34 is activated by amino acids and is involved in mediating the effect of amino acids on mTORC1 [68,69]. It is unknown if hVps34 signals through Rheb or if it affects directly mTORC1.

Cellular energy status also converges to mTOR. In response to energy starvation (low ATP level), mTORC1 activity is inhibited through the phosphorylation of TSC2 by AMPK (AMP-activated protein kinase). AMPK is activated by LKB1 (Liver kinase B1) that directly phosphorylates the activation loop and increases AMPK kinase activity [70]. It was proposed that AKT counteracts this effect by the preservation of nutrient uptake that sustain high levels of ATP and a low level of AMPK activity, leading to the inhibition of TSC2 and the activation of mTORC1 [71]. In response to energy starvation, cells also increase the mRNA levels of the hypoxia-inducible gene REDD1 (Regulated in development and DNA damage responses 1), which activates TSC2 and inhibits Rheb [72].Hypoxia has also an inhibitory effect on mTORC1 activity, which is in part mediated by the induction of REDD1 [73]. Transcriptional upregulation of REDD1 during hypoxia has been described to be dependent on the hypoxia-inducible transcription factor, HIF-1 [74]. Hypoxia can also inhibit mTORC1 independently of REDD1 and HIF-1, via the induction of energy stress. AMPK/TSC2/Rheb pathway is activated at low oxygen levels, resulting in mTORC1 inhibition [75].

In addition to activation by PI3K and AMPK, RAS/MAPK signalling has also been shown to trigger the activation of mTORC1 signalling. RAS proteins (H-, K- and N-RAS) function as a GDP/GTP-regulated switch and may have an important role as oncoproteins. In normal quiescent cells, RAS is GDP-bound and inactive. Upon stimulation by growth factors, hormones or cytokines, the activated GTP-bound form of RAS binds to and activates RAF kinase [76,77]. Upon activation, RAF phosphorylates and activates MEK, which activates ERK/RSK pathway. ERK phosphorylates both cytosolic and nuclear substrates leading to regulation of gene expression, cytoskeleton and metabolic remodelling [78,79]. ERK and RSK induce inhibitory phosphorylation of TSC2 at Ser664 and Ser1798 respectively, which promotes TSC1/TSC2 dissociation, which in turn leads to mTORC1 activation [80–82]. Interestingly, it was recently described that RSK also targets directly the mTORC1 complex by phosphorylating raptor, and thereby promotes mTORC1 kinase activity [83]. As tumour promoting phorbol esters and some growth factors activate mTORC1 signalling independently of AKT, phosphorylation of raptor by RSK might provide a mechanism to overcome the inhibitory effects of PRAS40. In addition, the ERK activated protein kinases MNK1 and MNK2 (MAPK-interacting protein kinase 1 and 2) directly phosphorylate eIF4E [84,85]. Together, these findings may indicate that the mitogens activated RAS-ERK-RSK signalling, in parallel with the PI3K-AKT pathway, contain several inputs to stimulate mTORC1 signalling.

Cytokines, such as TNFα (tumour necrosis factor α), can also activate mTORC1 [86,87]. It has been described that IKKβ (inhibitor of nuclear factor κB (NFκB) kinase β), a major downstream kinase in the TNFα signalling pathway, phosphorylates TSC1 at Ser487 and Ser511, leading to the inhibition of TSC1/TSC2 complex formation and mTORC1 activation [88]. Moreover, TNFα also signals to AKT [89]. Activated AKT induces IKKα, another major downstream kinase in the TNFα signalling pathway [90]. It has been described that IKKα associates with mTORC1 in an AKT dependent manner. Importantly, IKKα is required for the efficient induction of mTORC1 activity by AKT in cell lines [91].

Although mTORC1 activity is prone to multiple positive and negative regulations driven by extracellular growth factors and stress stimuli, mTORC2 regulation mechanisms remain largely unknown. In mammalian cells, mTORC2 phosphorylates AKT upon serum stimulation, namely growth factors such as insulin and IGF1 (insulin-like growth factor 1), suggesting that mTORC2 is regulated by the PI3K pathway [35,92]. Nevertheless, the mechanism by which insulin or other growth factors activate mTORC2 is unclear. A recent study advocates that growth factors may signal to mTORC2 via the TSC1/TSC2 complex. Huang and co-authors proposed that the TSC1/TSC2 complex, the upstream negative regulator of mTORC1, may also bind to and regulate mTORC2 activity by direct binding to mTORC2 [93]. Distinctive to the negative regulation of mTORC1, TSC1/TSC2 seems to regulate positively mTORC2 activity in a GAP-independent manner. The GTPase Rheb, which is a downstream of TSC1/TSC2 and activates mTORC1 [62], does not appear to operate upstream of mTORC2. Since TSC1/TSC2 GAP activity is not required for mTORC2 activation, it appears that it is independent of mTORC1 activation and of the negative feedback loop from mTORC1 and S6K1 to the upstream IRS [94].

Thus, the mTOR pathway can be activated by diverse exogenous stimuli, such as growth factors, nutrients, energy and stress signals, and essential signalling pathways, such as PI3K, MAPK and AMPK, in order to regulate several physiological events.

## 3. Physiological Roles of the mTOR Pathway in Control of Growth and Lifespan

Cell growth includes the time and space process of mass accumulation. In the presence of appropriate growth stimuli, cells upregulate macromolecular synthesis and thereby increase in size and mass. In response to stress, cells restrain macromolecular synthesis and enhance turnover of mass burden. Studies in yeast described that TOR plays two essential functions in the control of growth: when and where a cell grows (reviewed in [2]). When growth conditions are favourable, TOR is active and yeast cells maintain a robust rate of ribosome biogenesis, translation initiation, and nutrient import. Noteworthy, rapidly growing yeast cells treated with rapamycin, starved for nitrogen, or depleted of both TOR1 and TOR2 downregulate general protein synthesis and activate several stress-responsive transcription factors. Mutation of dTOR in *Drosophila* and rapamycin treatment in mammalian systems induces a significant reduction on cell size [70,95,96]. These results indicate that TOR is an important cell size regulator. Additionally, it has been suggested that S6K1 and 4EBP1, two of the well-characterized TOR targets, are key TOR pathway elements that mediate the regulation of cell size [96]. TOR2 also functions to regulate spatial aspects of yeast cell growth, by controlling the actin cytoskeleton [2]. In yeast, rapamycin-insensitive TORC2 controls the cell cycle-dependent polarization of the actin cytoskeleton. TORC2 signals to the actin cytoskeleton by activating a Rho1 GTPase switch. Upon activation, Rho1 interacts and activates PKC1, which in turn signals to the actin cytoskeleton via MAPK pathway [97,98]. mTORC2 also signals to the actin cytoskeleton, and although the direct targets of mTORC2 are unknown, this signalling may involve PKCα and the small GTPases Rho and Rac [12,34].

mTORC1 signalling controls transcription of many genes, some of which are involved in metabolic and biosynthetic pathways, as described in microarray experiments on rapamycin-treated mammalian cells [99]. mTOR also regulates nutrient-responsive transcription programs [100,101]. Furthermore, mTOR has been described to phosphorylate STAT1 and STAT3 (signal transducer and activator of transcription) [102] and to activate the nuclear receptor PPARγ, in a rapamycin-sensitive manner.

Cells must have mechanisms that regulate their growth and proliferation, by tight control of ribosomal biogenesis, which is an energy and nutrient-consuming process. Studies in both yeast and mammalian cells described that TOR regulates ribosome biogenesis at multiple levels, including transcription, rRNA processing, and translation, which can be inhibited by rapamycin or by nutrient starvation [103–105].

mTOR has also been described as a key signalling regulator of autophagy. Autophagy is a highly conserved eukaryotic intracellular homeostatic process carrying out degradation of cytoplasm components, including damaged or superfluous organelles, toxic protein aggregates, and intracellular pathogens in lysosomes [106,107]. Autophagy can be upregulated during metabolic, genotoxic or hypoxic stress conditions in order to ensure cell survival [106]. Inhibition of mTOR kinase by specific inhibitors, rapamycin or nutrient deprivation, induces activation of autophagy [107]. The role of mTOR in autophagy is conserved from yeast to mammals, and regulates the induction of the autophagy process [108]. In mammals this process may be mediated in part through mTOR-dependent phosphorylation of eEF2K (eukaryotic translation elongation factor 2 kinase), where mTOR inhibition leads to activation of eEF2K and induction of autophagy [109]. The release of amino acids from autophagic degradation leads to the reactivation of mTORC1 and to the restoration of the cellular lysosomal population [110].

mTORC1 also regulates translation via S6K1 and 4EBP1, as referred above [21,111].

TOR controls many aspects of cellular metabolism, such as amino acid biosynthesis and glucose homeostasis [112]. mTORC1 appears also to play an important role in adipogenesis; rapamycin treatment prevents adipocyte differentiation and lipid accumulation [113]. The mechanism by which mTOR controls adipogenesis might involve the nuclear receptor PPARγ that is responsible for the efficiency of energy storage [114], since its activity is inhibited by rapamycin treatment [113]. The regulation of fat metabolism by mTORC1 also involves signalling via S6K1. S6K1 mutant mice display reduced adipose tissue and a decrease in fat accumulation due to enhanced β oxidation [115].

Partial inhibition of TOR function in yeast, worms, and flies results in a significant lifespan increase of these organisms, possibly by mimicking calorie restriction (reviewed in [116]). It appears that during development, TOR controls primarily growth, whereas in the adult, when there is a relative slow growth, TOR controls aging and other aspects of nutrient-related physiology. Recent studies corroborate the role of calorie restriction also in the lifespan extension of mammals [117,118]. Rapamycin, an mTORC1 inhibitor, is the only pharmacological agent that has been described to mimic calorie restriction and extended lifespan [118].

Hence, the mTOR pathway is a central coordinator of fundamental biological events, playing a key role in cell growth and size, regulation of actin cytoskeleton, gene transcription, ribosome biogenesis, mRNA translation, cell survival and lifespan.

## 4. The mTOR Pathway in Cancer

Given the key role of mTOR in cell growth and metabolism, it is predictable the existence of an association between mTOR pathway activity and pathological states, including cancer.

Activation of the mTOR signalling is involved in some of the cancer hallmarks described by Hanahan and Weinberg [119]. In a number of *in vitro* cell-lines and *in vivo* murine xenograft models, aberrant mTOR pathway activation through oncogene stimulation or loss of tumour suppressors contributes to tumour growth, angiogenesis and metastasis [56]. Mutations in mTOR gene that confer constitutive activation of mTOR signalling, even under nutrient starvation conditions, have been identified in a few human cancers, although not clearly linked to tumour development [120]. In spite of this, as summarized in Table 1, the signalling components upstream and downstream of mTORC1 are frequently altered in human tumours.

Upstream, PI3K/AKT signalling is deregulated through a variety of mechanisms, including overexpression or activation of growth factor receptors, such as HER-2 (human epidermal growth factor receptor 2) and IGFR (insulin-like growth factor receptor), mutations in *PI3K* and mutations/amplifications of *AKT* [121–123].

PTEN, the negative regulator of PI3K signalling, decreases its expression in many cancers, and may be downregulated through several mechanisms, including mutation, loss of heterozygosity, methylation, aberrant expression of regulatory microRNA, and protein instability [124,125].

mTOR downstream effectors S6K1, 4EBP1 and eIF4E are implicated in cellular transformation, and their overexpression has been linked to poor cancer prognosis [127,137,150,151]. Activated mTOR signalling is also related to the development of syndromes, including Cowden’s syndrome (*PTEN* mutations), Peutz-Jeghers syndrome (*LKB1* mutations) and tuberous sclerosis (*TSC1/2* mutations) [143,152–154]. These syndromes, in which the patients develop benign tumours that contain architecturally disorganized but well differentiated cells, affect a wide variety of tissues, comprising brain, skin, kidneys, heart, lung, and the gastrointestinal tract. Though benign, these syndromes may progress to malignancy.

Thus, mTOR signalling is activated in conditions of proliferation deregulation and in many cancer types. Deregulation of multiple elements of the mTOR pathway (*PI3K* amplification/mutation, *PTEN* loss of function, AKT overexpression, and S6K1, 4EBP1 and eIF4E overexpression) has been reported in cancer, such as breast, ovarian, renal, colon and head and neck cancers. Taken together, these data underscore the importance of mTOR signalling in cancer and reinforce the importance of considering mTOR targeting in cancer therapy.

## 5. The mTOR Pathway in Melanoma

Activation of the AKT/mTOR signalling pathways plays a role in the initiation of melanocyte tumours by modulating the extracellular signals that control cell growth, proliferation and apoptosis [138,155].

Loss of *PTEN*, the negative regulator of PI3K pathway, was described in 30–50% of melanomas, and correlates with melanoma progression and with shorter 5-year survival [156,157]. Studies in melanoma cell lines and primary or metastatic melanomas described that disruption of *PTEN* by allelic loss or mutation contributes to the pathogenesis of malignant melanoma [158,159]. Loss of *PTEN* and *RAS* activation seems comparable in their ability to increase oncogenic signalling through PI3K pathway [160], due to the coexistence of *PTEN* somatic mutations in melanoma harbouring *BRAF* mutations but not with *NRAS* [161]. *AKT* gene amplification, owing to copy number increase in the long arm of chromosome 1 and activating mutation, was also described in cutaneous melanomas [156,162]. Immunohistochemistry studies described AKT overexpression in 60% of melanomas, differing from common and dysplastic nevi that do not exhibit significant AKT expression [163]. Amongst the three AKT isoforms, AKT3 is the isoform more frequently deregulated in melanoma cells [164]. In melanoma, the increased pAKT expression associates with tumour progression and shorter survival of patients [164,165]. Notably, AKT activity seems to cooperate in *BRAF^V600E^*-mediated model of melanoma development [166]. PRAS40, a substrate of the AKT, was described to be downregulated in melanoma [167]. Concordantly, results from our group indicate that the mTOR pathway is activated in cutaneous melanoma, displaying different levels of activation in different histological subtypes, and relate with MAPK pathway activation. Our results suggest an association between higher mTOR effectors expression and worse prognosis as well as with the presence of *BRAF* mutations [138].

There is also evidence that the AKT/mTOR pathway is altered in uveal melanoma [168,169]. PTEN displays decreased expression in aggressive tumours [170] and expression of AKT phosphorylated at Ser473 was proposed as a marker of worse prognosis [168]. Furthermore, results from our group also indicate that the mTOR pathway is activated in ocular melanoma and is related with MAPK pathway activation. Our results show that the pathway activation seems to be higher in conjunctival than in uveal melanomas and this activation seems to be associated with worse prognosis, especially in conjunctival melanomas. Besides, higher expression of pAKT Thr308 was found in metastatic uveal melanoma [169].

Overall, the alterations in major components of the MAPK, such as *BRAF* and *NRAS* mutations, and mTOR pathways, *PTEN* loss and AKT overexpression, seem to have substantial influence in melanoma progression, being both pathways linked to survival and chemoresistence in melanoma [3,171,172].

## 6. mTOR Pathway Inhibitors in Cancer Therapy

mTOR inhibitors can be grouped in two classes: rapamycin and rapamycin analogues that are allosteric inhibitors of mTORC1 and the small molecules that are mTOR kinase inhibitors.

Rapamycin was first isolated from the bacterium *Streptomyces hygroscopicus*, which was found in a soil sample taken from Easter Island, as a fungicide and subsequently discovered to have potent immunosuppressive and anti-tumour properties [173–175]. As an immunosuppressive drug, rapamycin (rapamune, sirolimus) was approved by FDA (USA Food and Drug Administration) in 1999 for prevention of renal allograft rejection [176]. Subsequent studies described that rapamycin can also act as a cytostatic agent, slowing or arresting growth of cell lines derived from different tumour types such as rhabdomyosarcoma [177], glioblastoma [178], small cell lung cancer [179], osteosarcoma [180], pancreatic cancer [181], breast cancer [182], prostate cancer [183], and B-cell lymphoma [184]. In addition to direct anti-tumour effects, rapamycin also inhibits cell proliferation, survival and angiogenesis [185,186].

Several derivatives of rapamycin (sirolimus, Wyeth, Madison, NJ, USA), with more favourable pharmacokinetic and solubility properties, have been synthesized, such as CCI-779 (temsirolimus, Wyeth, Madison, NJ, USA), RAD001 (everolimus, Novartis, Novartis, Basel, Switzerland), AP23573 (deforolimus, ARIAD, Cambridge, MA, USA), 32-deoxorapamycin (SAR943) or ABT-578 (zotarolimus, Abbott Laboratories, Abbott Park, IL, USA) (Table 2). Like rapamycin, these rapamycin analogues form a complex with the intracellular receptor FKBP12. This complex binds to mTOR and inhibits mTORC1 downstream signalling, detected by the suppression of S6K1 and 4EBP1 phosphorylation [187,188]. The FKBP12-rapamycin complex cannot bind directly to mTORC2, although prolonged treatments can disturb mTORC2 assembly and inhibit the phosphorylation of its downstream substrate AKT on Ser473 [34,39].

Rapamycin and its analogues temsirolimus, everolimus and deforolimus are currently being evaluated in clinical trials for cancer treatment (Table 2) [189]. In preclinical studies they were described to carry out antiproliferative activity in a variety of cancers, and there are clinical studies reporting encouraging results in a subset of cancers [190–192]. Remarkably, a high objective response rate was reported with treatment with rapamycin analogues of several tumour types. Phase II studies with everolimus achieved an objective response rate of 47%, 30% and 12%, with median duration of response of 7.2, 5.7 and 13.1 months in Hodgkin lymphoma, non-Hodgkin’s lymphoma and breast cancer, respectively [193–195]. Phase II/III studies with temsirolimus achieved an objective response rate of 4 to 14% and 22%, with median duration of response of 4.3 to 5.1 and 4.8 months in endometrial cancer and mantle-cell lymphoma, respectively [196,197].

However, several studies also suggested that the antiproliferative effects of the analogues are variable in cancer cells due to failure of mTORC2 inhibition in some tumour types. The specific inhibition of mTORC1 by RAD001 might induce upstream receptor tyrosine kinase signalling and AKT upregulation, leading to the attenuation of its therapeutic effects [198]. Thus, the combination therapy or a dual-specificity agent that targets both mTOR function and AKT activation may improve anti-tumour activity.

Noteworthy, temsirolimus and everolimus were approved by the FDA for the treatment of renal cell carcinoma (RCC) (http://www.fda.gov). mTOR inhibition seems to downregulate HIF, which is frequently overexpressed in RCC, due to loss of function of VHL (Von Hippel-Lindau) gene [199,200]. Likewise, everolimus was approved by FDA for the treatment of progressive endocrine tumours of pancreatic origin (PNET) in patients with unresectable, locally advanced or metastatic disease and for patients with subependymal giant cell astrocytoma (SEGA) associated with tuberous sclerosis (TS) (http://www.fda.gov).

A new generation of mTOR inhibitors, which bind to the ATP-binding site of mTOR and inhibit the catalytic activity of mTORC1 and mTORC2, were developed (Table 2).

Distinct from rapamycin analogues, these molecules block both mTORC1-dependent phosphorylation of S6K1 and mTORC2-dependent phosphorylation of the AKT Ser473 residue. The anticancer efficacy of these inhibitors reported in preclinical evaluation has been superior to rapamycin analogues. This was related to a more effective blocking of cell proliferation, 4EBP1 phosphorylation and protein translation, compared to rapamycin [201,202]. Two active-site inhibitors of mTOR, PP242 and PP30, which inhibit insulin-stimulated phosphorylation of AKT at Ser473, were reported as having potent inhibitory effects on protein synthesis and cell proliferation [201]. Torin1, another selective ATP-competitive mTOR inhibitor, which directly inhibits both mTORC1 and mTORC2, also seems to inhibit cell growth and proliferation more effectively than rapamycin [202]. Several selective mTOR inhibitors are in development stage and more studies are warranted to further evaluate the efficacy of these agents in the treatment of cancers affected by hyperactive PI3K/mTOR pathway.

Dual PI3K-mTOR inhibitors are also being developed. This class of inhibitors includes XL-765 (Sanofi-Aventis/Exelixis Inc) [203], which is undergoing phase I clinical trials for the treatment of solid tumours and gliomas (NCT00485719, NCT00704080 and NCT00777699), PI-103 [204] and NVP-BEZ235 (Novartis AG), which is undergoing phase I/II trials for the treatment of advanced solid tumours and metastatic breast cancer [205]. These compounds were reported to prevent the activity of PI3K-mTOR axis biomarkers more effectively than rapamycin, by inhibiting both mTORC1 and mTORC2. The dual PI3K-mTOR inhibitors are still in phase I/II clinical trials.

So far and for most tumour types, mTOR inhibitors have been reported to predominantly lead to disease stabilization rather than tumour regression. Assuming these results, targeted therapies for mTOR may be used in combination therapy, aiming to induce a cytotoxic rather than a cytostatic response and subsequent tumour regression. mTOR inhibitors have been described to be additive or synergistic with conventional chemotherapy agents, such as paclitaxel, carboplatin, cisplatin, vinorelbine, doxorubicin, and camptothecin [178,213–215]. Compared to single agent therapy, the combination of rapamycin with chemotherapy enhances apoptosis *in vitro* and enhance anti-tumour efficacy *in vivo*. Clinical trials to evaluate the efficacy of rapamycin and analogues in combination with chemotherapeutic agents are in progress.

Rapamycin analogues are also being tested in combination with EGFR or HER-2 inhibitors. Early trials of EGFR inhibitors combined with analogues in glioblastoma patients did not disclose any positive results and lung cancer patients resistant to EGFR inhibitors showed toxic effects that required discontinuation or dose reductions in some patients [216,217]. Trials combining hormonal therapies with mTOR inhibitors are been performed in breast cancer, since resistance to hormonal therapy has been associated with overactivation of the mTOR pathway [218]. Phase I/II trials with trastuzumab combined with everolimus in Her-2+ metastatic breast cancer that progressed on trastuzumab therapy reported clinical benefit and restore trastuzumab sensitivity [219]. Phase I and II trials of mTOR inhibitors in combination with erlotinib, gefitinib or cetuximab are ongoing.

Since mTOR inhibitors downregulate HIF and VEGF, combination of temsirolimus or everolimus with bevacizumab, sorafenib or sunitinib are being tested in clinical trials. The combination with bevacizumab seems to be better tolerated and more effective than combinations using small molecules targeting VEGFR [220]. The combination of temsirolimus with sorafenib, which targets RAF-1 and other kinases in addition to VEGFR, required a 50% reduction of the single-agent dose of sorafenib to achieve an acceptable range of toxic effects [221]. Temsirolimus combined with sunitinib, which also inhibits VEGFR and other kinases, caused excessive toxic effects [222]. Trials evaluating temsirolimus combined with bevacizumab, temsirolimus combined with sorafenib and compared with bevacizumab alone are ongoing in a randomized phase II study of untreated patients with metastatic RCC (NCT00378703). Besides, an ongoing phase III trial is evaluating the combination of bevacizumab and temsirolimus as second-line of therapy for RCC (NCT00631371). Other drug combinations being tested are directed to the feedback loops triggered by mTORC1 inhibition. As rapamycin analogues treatment leads to AKT activation through IRS-1 signalling [223], mTORC2 phosphorylation of AKT Ser473, or activation of the MAPK pathway [63], the combinations with inhibitors targeting these pathways were evaluated. Studies of analogues combined with IGF-1 inhibitors or MAPK2 inhibitors reported synergistic effects [224–226]. Phase I clinical trials are in progress to evaluate the safety and tolerability of these combination therapies.

Regarding melanoma, rapamycin, may also synergistically increase apoptosis and chemosensitivity in melanoma cells [227,228]. The anti-tumour effects of rapamycin seem to be enhanced when combined with MAPK pathway inhibitors and PI3K inhibitors. The PI3K inhibitor LY294002 abrogates the AKT phosphorylation induced by mTORC1 inhibition [229], while the MAPK pathway inhibitor sorafenib downregulates the expression of the anti-apoptotic proteins Mcl-1 and Bcl-2 [225,227]. Temsirolimus combined with cisplatin efficiently induced regression of melanomas in SCID mice, being both inhibitors much less effective when applied as single agents [230]. Although phase II clinical trials with mTOR inhibitors alone yield minor responses and/or high toxicity in melanoma patients (reviewed in ref. [231]), phase II clinical trials combining temsirolimus and sorafenib are ongoing (NCT00349206). In addition, as vemurafenib, a specific BRAF^V600E^ inhibitor, was approved by the FDA for the treatment of patients with unresectable or metastatic melanoma with BRAF^V600E^ mutation, a trial combining vemurafenib with mTOR inhibitors may be valuable.

Thus, mTOR is an appealing therapeutic target. The rapamycin analogues deforolimus, everolimus and temsirolimus, are being evaluated in clinical trials for treating multiple cancers, alone or in combination with inhibitors of other pathways. Importantly, temsirolimus and everolimus were recently approved by the FDA for the treatment of renal cell carcinoma, PNET and giant cell astrocytoma. Small molecules that inhibit mTOR kinase activity and dual PI3K-mTOR inhibitors are also being developed.

## 7. mTOR Therapy Predictive Biomarkers

A major challenge for the development of cancer therapy is the identification of predictive biomarkers of efficacy. There are no known predictive biomarkers for the efficacy or resistance in cancer of mTOR inhibitors. Activation of PI3K signalling, through AKT activation, PTEN deletion, growth factor stimulation or aberrant growth factor receptor signalling, may indicate potential sensitivity of tumours to mTOR inhibition [232]. Thus, the differential expression of mTOR pathway proteins (PTEN and active forms of AKT and S6) may be possibly predictive markers for tumour response to mTOR inhibition, as described in glioblastoma, prostate and breast cancer cell lines [233,234]. Nevertheless, these predictors of response have been proposed based only on preclinical data and in specific types of cancer, and have not been clinically validated, with the exception of the loss of PTEN expression, that was used in clinical trials as a marker to evaluate glioblastoma sensitivity to rapamycin treatment [235]. Moreover, there is a considerable variation in the therapeutic benefits detected in patients harbouring tumours predicted to be responders to mTOR inhibition, because the genetic context in which the altered phenotype occurs may also be important for the patient response to therapy.

Therefore, there is an emergent need to identify predictive markers of response that may be useful to prospectively select patients bearing tumours which may respond and benefit from mTOR inhibition therapies.

## 8. Conclusions

In the last years, a significant progress has been achieved in understanding the mTOR signalling pathway. mTOR has been suggested to play a key role in several normal biological processes as well as in disease. It is known that mTOR forms two multiprotein complexes, mTORC1 and mTORC2, which have distinct physiological functions. Deregulation of multiple elements of the mTOR pathway has been reported in many types of cancers.

Most of the research on the mTOR signalling pathway has been focused on using rapamycin, which blocks mainly mTORC1 activity. Novel compounds that inhibit mTORC1 and mTORC2 are likely to reveal as yet undiscovered components and complexities of the mTOR pathway. Eventually, the study of the mTOR pathway may bring novel insights into mTOR biology, and also assist in the development of more effective therapeutic strategies for treating mTOR-related diseases, particularly cancer. mTOR is now considered a substantiated target in the treatment of cancer. The major limitation for the development of mTOR inhibition therapy is the absence of predictive biomarkers of efficacy and its resistance mechanisms in cancer. The lack of routine genotyping of tumours is also part of the limitations in establishing predictive biomarkers for the use of mTOR inhibitors across the spectrum of human tumours. Stratification of patients and selection of drug combination therapies may enhance the efficacy of mTOR inhibition, leading to a more effective and personalized cancer therapy.

## Figures and Tables

**Table 1:** Proto-oncogenes and tumour suppressor genes linked to the mTOR pathway.

Proto-oncogenes	Alterations described	References
*AKT*	*AKT* is amplified in a subset of human cancers, such as breast and ovarian cancers.	[126]
*4EBP1*	4EBP1 expression was found to be associated with poor prognosis in several human tumours, such as breast, colon, ovarian and prostate cancers. The phosphorylation of 4EBP1 was also found to be associated with chemoresistance in ovarian cancer.	[127–129]
*eIF4E*	Ectopic overexpression of eIF4E can transform cells *ex vivo* and *in vivo*. eIF4E is overexpressed in many human tumours, such as breast, colon, and head and neck cancers, non-Hodgkin’s lymphomas, and chronic and acute myelogenous leukemias.	[130,131]
*PI3K*	High *PI3K* activity was implicated in cell transformation and tumour progression and described in several human cancers, such as ovarian, gastrointestinal, breast and prostate cancers.	[126, 132–134]
*Rheb*	Rheb overexpression is described in many tumour cells, and Rheb upregulation is critical for squamous carcinoma and associates with poor prognosis in breast and head and neck cancers.	[135,136]
*S6K1*	S6K1 is overexpressed in in lung and ovary cancers and its expression correlates with poor prognosis in breast, kidney and hepatocellular carcinomas.	[137–142]
**Tumour suppressor genes**		
*LKB1*	Individuals with mutations in *LKB1* develop Peutz-Jeghers syndrome, which includes the occurrence of gastrointestinal tract hamartomas.	[111,143]
*PTEN*	Loss of *PTEN* function has been described in a large proportion of advanced human cancers, such as melanoma, breast, prostate and renal cancers. Individuals with inherited mutations in *PTEN* develop hamartoma tumour syndromes (Cowden disease, Bannayan-Riley-Ruvalcaba syndrome, Proteus syndrome, Lhermitte-Duclos disease) and are at higher risk for developing several cancers.	[124,132, 143–146]
*TSC1/TSC2*	Patients with mutations in *TSC1* or *TSC2* develop tuberous sclerosis complex (TSC), a syndrome that includes the development of hamartomas in many organs. Mutations in *TSC2* may also lead to the development of Lymphangioleiomyomatosis (LAM).	[147–149]

**Table 2:** mTOR inhibitors in clinical trials.

mTOR inhibitors	Mechanism of action	References
**Rapamycin and analogues**		
Deforolimus	Binding to the immunophilin FKBP12 Partial mTORC1 inhibitor Cell-type specific mTORC2 inhibitor	[206]
Everolimus	Binding to the immunophilin FKBP12 Partial mTORC1 inhibitor Cell-type specific mTORC2 inhibitor	[206]
Sirolimus	Binding to the immunophilin FKBP12 Partial mTORC1 inhibitor Cell-type specific mTORC2 inhibitor	[206]
Temsirolimus	Binding to the immunophilin FKBP12 Partial mTORC1 inhibitor Cell-type specific mTORC2 inhibitor	[206]
**Small molecule inhibitors of kinases**		
AZD8055	ATP competitive inhibitor of mTOR	[207]
Ku-0063794	Specific mTORC1 and mTORC2 inhibitor	[208]
PP242	mTOR kinase inhibitor	[201]
PP30	mTOR kinase inhibitor	[201]
Torin1	mTOR kinase inhibitor	[202]
WYE-354	ATP competitive inhibitor of mTOR	[209]
**mTOR and PI3K dual-specificity inhibitors**		
NVP-BEZ235	ATP-competitive inhibitor of PI3K and mTOR	[205]
PI-103	ATP competitive inhibitor of DNA-PK, PI3K and mTOR	[210]
PKI-179, PKI-587	ATP competitive inhibitor of DNA-PK, PI3K and mTOR	[211,212]
XL765	ATP-competitive inhibitor of DNA-PK, PI3K and mTOR	[203]

## Non-Standard Abbreviations

4EBP1: Eukaryotic translation initiation factor 4E-binding protein 1
AMPK: 5′ Adenosine monophosphate-activated protein kinase
AKT/PKB: Protein kinase B
Bcl-2: B-cell lymphoma 2
BRAF: v-RAF murine sarcoma viral oncogene homolog B
DEPTOR: DEP domain-containing mTOR-interacting protein
eEF2K: Eukaryotic elongation factor-2 kinase
EGFR: Epidermal growth factor receptor
eIF4E: Eucaryotic translation initiation factor 4E
ERK: Extracellular regulated MAP kinase
FDA: Food and drug administration
FOXO: Forkhead transcriptor factor
GAP: Gtpase-activating protein
GNAQ: Guanine nucleotide-binding protein G(q) subunit alpha
GSK3: Glycogen synthase kinase 3
HIF-1: Hypoxia-inducible factor 1
Hsp**7**0: Heat shock protein **7**0-alpha
IRS-1: Insulin receptor substrate 1
KIT: v-kit Hardy-Zuckerman 4 feline sarcoma viral oncogene homolog
MAPK: Mitogen-activated protein kinase
MEK: Mitogen activated protein kinase kinase
MITF: Microphthalmia-associated transcription factor
mLST8: Mammalian lethal with SEC13 protein 8
mSin1: Mammalian stress activated protein kinase interacting protein 1
mTOR: Mammalian target of rapamycin
mTORC1/2: mTOR complex 1/2
MYC: Myelocytomatosis viral oncogene
NRAS: Neuroblastoma RAS viral (v-ras) oncogene homolog
PARP: Poly (ADP-ribose) polymerase
PDK 1: Phosphoinositide-dependent kinase 1
PI3K: Phosphatidylinositol 3-kinase
PIP2/ PtdIns(4,5)P2: Phosphatidylinositol-4,5-bisphosphate
PIP3/PtdIns(3,4,5)P3: Phosphatidylinositol-3,4,5-triphosphate
PKC: Protein kinase C
PRAS40: Proline rich Akt substrate 40
PRR5/Protor: Proline rich protein 5 / protein observed with rictor
PTEN: Phosphatase and tensin homologue deleted on chromosome ten
Raptor: Regulatory associated protein of mTOR
RAS: Rat sarcoma virus oncogene
REDD1: Protein regulated in development and DNA damage response 1
Rheb: Ras homolog enriched in brain
Rictor: Rapamycin insensitive companion of mTOR
RSK: p90 ribosomal S6 kinase
RTK: Receptor tyrosine kinase
S6K: S6 kinase
STAT: Signal transducer and activator of transcription
TSC 1/2: Tuberous sclerosis complex 1/2
VEGFR: Vascular Endothelial Growth Factor Receptor

## References

[1] Huang S., Houghton P.J. (2003). Targeting mTOR signaling for cancer therapy. Curr. Opin. Pharmacol.

[2] Wullschleger S., Loewith R., Hall M.N. (2006). TOR signaling in growth and metabolism. Cell.

[3] Guertin D.A., Sabatini D.M. (2007). Defining the role of mTOR in cancer. Cancer Cell.

[4] Heitman J., Movva N.R., Hall M.N. (1991). Targets for cell cycle arrest by the immunosuppressant rapamycin in yeast. Science.

[5] Sabers C.J., Martin M.M., Brunn G.J., Williams J.M., Dumont F.J., Wiederrecht G., Abraham R.T. (1995). Isolation of a protein target of the FKBP12-rapamycin complex in mammalian cells. J. Biol. Chem.

[6] Sabatini D.M., Erdjument-Bromage H., Lui M., Tempst P., Snyder S.H. (1994). RAFT1: A mammalian protein that binds to FKBP12 in a rapamycin-dependent fashion and is homologous to yeast TORs. Cell.

[7] Brown E.J., Albers M.W., Shin T.B., Ichikawa K., Keith C.T., Lane W.S., Schreiber S.L. (1994). A mammalian protein targeted by G1-arresting rapamycin-receptor complex. Nature.

[8] Keith C.T., Schreiber S.L. (1995). PIK-related kinases: DNA repair, recombination, and cell cycle checkpoints. Science.

[9] Kunz J., Henriquez R., Schneider U., Deuter-Reinhard M., Movva N.R., Hall M.N. (1993). Target of rapamycin in yeast, TOR2, is an essential phosphatidylinositol kinase homolog required for G1 progression. Cell.

[10] Zhou H., Huang S. (2010). The complexes of mammalian target of rapamycin. Curr. Protein Pept. Sci.

[11] Loewith R., Jacinto E., Wullschleger S., Lorberg A., Crespo J.L., Bonenfant D., Oppliger W., Jenoe P., Hall M.N. (2002). Two TOR complexes, only one of which is rapamycin sensitive, have distinct roles in cell growth control. Mol. Cell.

[12] Sarbassov D.D., Ali S.M., Kim D.H., Guertin D.A., Latek R.R., Erdjument-Bromage H., Tempst P., Sabatini D.M. (2004). Rictor, a novel binding partner of mTOR, defines a rapamycin-insensitive and raptor-independent pathway that regulates the cytoskeleton. Curr. Biol.

[13] Hara K., Maruki Y., Long X., Yoshino K., Oshiro N., Hidayat S., Tokunaga C., Avruch J., Yonezawa K. (2002). Raptor, a binding partner of target of rapamycin (TOR), mediates TOR action. Cell.

[14] Kim D.H., Sarbassov D.D., Ali S.M., King J.E., Latek R.R., Erdjument-Bromage H., Tempst P., Sabatini D.M. (2002). mTOR interacts with raptor to form a nutrient-sensitive complex that signals to the cell growth machinery. Cell.

[15] Sancak Y., Thoreen C.C., Peterson T.R., Lindquist R.A., Kang S.A., Spooner E., Carr S.A., Sabatini D.M. (2007). PRAS40 is an insulin-regulated inhibitor of the mTORC1 protein kinase. Mol. Cell.

[16] Peterson T.R., Laplante M., Thoreen C.C., Sancak Y., Kang S.A., Kuehl W.M., Gray N.S., Sabatini D.M. (2009). DEPTOR is an mTOR inhibitor frequently overexpressed in multiple myeloma cells and required for their survival. Cell.

[17] Gwinn D.M., Shackelford D.B., Egan D.F., Mihaylova M.M., Mery A., Vasquez D.S., Turk B.E., Shaw R.J. (2008). AMPK phosphorylation of raptor mediates a metabolic checkpoint. Mol Cell.

[18] Kim D.H., Sarbassov D.D., Ali S.M., Latek R.R., Guntur K.V., Erdjument-Bromage H., Tempst P., Sabatini D.M. (2003). GβL, a positive regulator of the rapamycin-sensitive pathway required for the nutrient-sensitive interaction between raptor and mTOR. Mol. Cell.

[19] Guertin D.A., Stevens D.M., Thoreen C.C., Burds A.A., Kalaany N.Y., Moffat J., Brown M., Fitzgerald K.J., Sabatini D.M. (2006). Ablation in mice of the mTORC components raptor, rictor, or mLST8 reveals that mTORC2 is required for signaling to Akt-FOXO and PKCα, but not S6K1. Dev. Cell.

[20] Zeng Z., Sarbassov dos D., Samudio I.J., Yee K.W., Munsell M.F., Ellen Jackson C., Giles F.J., Sabatini D.M., Andreeff M., Konopleva M. (2007). Rapamycin derivatives reduce mTORC2 signaling and inhibit AKT activation in AML. Blood.

[21] Hay N., Sonenberg N. (2004). Upstream and downstream of mTOR. Genes Dev.

[22] Choi J.H., Bertram P.G., Drenan R., Carvalho J., Zhou H.H., Zheng X.F. (2002). The FKBP12-rapamycin-associated protein (FRAP) is a CLIP-170 kinase. EMBO Rep.

[23] Redpath N.T., Foulstone E.J., Proud C.G. (1996). Regulation of translation elongation factor-2 by insulin via a rapamycin-sensitive signalling pathway. EMBO J.

[24] Seidel E.R., Ragan V.L. (1997). Inhibition by rapamycin of ornithine decarboxylase and epithelial cell proliferation in intestinal IEC-6 cells in culture. Br. J. Pharmacol.

[25] Azpiazu I., Saltiel A.R., DePaoli-Roach A.A., Lawrence J.C. (1996). Regulation of both glycogen synthase and PHAS-I by insulin in rat skeletal muscle involves mitogen-activated protein kinase-independent and rapamycin-sensitive pathways. J. Biol. Chem.

[26] Hudson C.C., Liu M., Chiang G.G., Otterness D.M., Loomis D.C., Kaper F., Giaccia A.J., Abraham R.T. (2002). Regulation of hypoxia-inducible factor 1α expression and function by the mammalian target of rapamycin. Mol. Cell Biol.

[27] Huffman T.A., Mothe-Satney I., Lawrence J.C. (2002). Insulin-stimulated phosphorylation of lipin mediated by the mammalian target of rapamycin. Proc. Natl. Acad. Sci. USA.

[28] Parekh D., Ziegler W., Yonezawa K., Hara K., Parker P.J. (1999). Mammalian TOR controls one of two kinase pathways acting upon nPKCδ and nPKCɛ. J. Biol. Chem.

[29] Peterson R.T., Desai B.N., Hardwick J.S., Schreiber S.L. (1999). Protein phosphatase 2A interacts with the 70-kDa S6 kinase and is activated by inhibition of FKBP12-rapamycinassociated protein. Proc. Natl. Acad. Sci. USA.

[30] Huang S., Liu L.N., Hosoi H., Dilling M.B., Shikata T., Houghton P.J. (2001). p53/p21(CIP1) cooperate in enforcing rapamycin-induced G(1) arrest and determine the cellular response to rapamycin. Cancer Res.

[31] Nourse J., Firpo E., Flanagan W.M., Coats S., Polyak K., Lee M.H., Massague J., Crabtree G.R., Roberts J.M. (1994). Interleukin-2-mediated elimination of the p27Kip1 cyclin-dependent kinase inhibitor prevented by rapamycin. Nature.

[32] Usui I., Haruta T., Iwata M., Takano A., Uno T., Kawahara J., Ueno E., Sasaoka T., Kobayashi M. (2000). Retinoblastoma protein phosphorylation via PI 3-kinase and mTOR pathway regulates adipocyte differentiation. Biochem. Biophys. Res. Commun.

[33] Yokogami K., Wakisaka S., Avruch J., Reeves S.A. (2000). Serine phosphorylation and maximal activation of STAT3 during CNTF signaling is mediated by the rapamycin target mTOR. Curr. Biol.

[34] Jacinto E., Loewith R., Schmidt A., Lin S., Ruegg M.A., Hall A., Hall M.N. (2004). Mammalian TOR complex 2 controls the actin cytoskeleton and is rapamycin insensitive. Nat. Cell Biol.

[35] Frias M.A., Thoreen C.C., Jaffe J.D., Schroder W., Sculley T., Carr S.A., Sabatini D.M. (2006). mSin1 is necessary for Akt/PKB phosphorylation, and its isoforms define three distinct mTORC2s. Curr. Biol.

[36] Pearce L.R., Huang X., Boudeau J., Pawlowski R., Wullschleger S., Deak M., Ibrahim A.F., Gourlay R., Magnuson M.A., Alessi D.R. (2007). Identification of Protor as a novel Rictor-binding component of mTOR complex-2. Biochem. J.

[37] Martin J., Masri J., Bernath A., Nishimura R.N., Gera J. (2008). Hsp70 associates with Rictor and is required for mTORC2 formation and activity. Biochem. Biophys. Res. Commun.

[38] Hresko R.C., Mueckler M. (2005). mTOR.RICTOR is the Ser473 kinase for Akt/protein kinase B in 3T3-L1 adipocytes. J. Biol. Chem.

[39] Sarbassov D.D., Ali S.M., Sengupta S., Sheen J.H., Hsu P.P., Bagley A.F., Markhard A.L., Sabatini D.M. (2006). Prolonged rapamycin treatment inhibits mTORC2 assembly and Akt/PKB. Mol. Cell.

[40] Um S.H., D’Alessio D., Thomas G. (2006). Nutrient overload, insulin resistance, and ribosomal protein S6 kinase 1, S6K1. Cell Metab.

[41] Alessi D.R., James S.R., Downes C.P., Holmes A.B., Gaffney P.R., Reese C.B., Cohen P. (1997). Characterization of a 3-phosphoinositide-dependent protein kinase which phosphorylates and activates protein kinase Bα. Curr. Biol.

[42] Stokoe D., Stephens L.R., Copeland T., Gaffney P.R., Reese C.B., Painter G.F., Holmes A.B., McCormick F., Hawkins P.T. (1997). Dual role of phosphatidylinositol-3,4,5-trisphosphate in the activation of protein kinase B. Science.

[43] Sarbassov D.D., Guertin D.A., Ali S.M., Sabatini D.M. (2005). Phosphorylation and regulation of Akt/PKB by the rictor-mTOR complex. Science.

[44] Stambolic V., Suzuki A., de la Pompa J.L., Brothers G.M., Mirtsos C., Sasaki T., Ruland J., Penninger J.M., Siderovski D.P., Mak T.W. (1998). Negative regulation of PKB/Akt-dependent cell survival by the tumor suppressor PTEN. Cell.

[45] Inoki K., Li Y., Zhu T., Wu J., Guan K.L. (2002). TSC2 is phosphorylated and inhibited by Akt and suppresses mTOR signalling. Nat. Cell Biol.

[46] Inoki K., Li Y., Xu T., Guan K.L. (2003). Rheb GTPase is a direct target of TSC2 GAP activity and regulates mTOR signaling. Genes Dev.

[47] Long X., Lin Y., Ortiz-Vega S., Yonezawa K., Avruch J. (2005). Rheb binds and regulates the mTOR kinase. Curr. Biol.

[48] Takahashi K., Nakagawa M., Young S.G., Yamanaka S. (2005). Differential membrane localization of ERas and Rheb, two Ras-related proteins involved in the phosphatidylinositol 3-kinase/mTOR pathway. J. Biol. Chem.

[49] Buerger C., DeVries B., Stambolic V. (2006). Localization of Rheb to the endomembrane is critical for its signaling function. Biochem. Biophys. Res. Commun.

[50] Kovacina K.S., Park G.Y., Bae S.S., Guzzetta A.W., Schaefer E., Birnbaum M.J., Roth R.A. (2003). Identification of a proline-rich Akt substrate as a 14-3-3 binding partner. J Biol Chem.

[51] Lim H.K., Choi Y.A., Park W., Lee T., Ryu S.H., Kim S.Y., Kim J.R., Kim J.H., Baek S.H. (2003). Phosphatidic acid regulates systemic inflammatory responses by modulating the Akt-mammalian target of rapamycin-p70 S6 kinase 1 pathway. J. Biol. Chem.

[52] Nojima H., Tokunaga C., Eguchi S., Oshiro N., Hidayat S., Yoshino K., Hara K., Tanaka N., Avruch J., Yonezawa K. (2003). The mammalian target of rapamycin (mTOR) partner, raptor, binds the mTOR substrates p70 S6 kinase and 4E-BP1 through their TOR signaling (TOS) motif. J. Biol. Chem.

[53] Schalm S.S., Fingar D.C., Sabatini D.M., Blenis J. (2003). TOS motif-mediated raptor binding regulates 4E-BP1 multisite phosphorylation and function. Curr. Biol.

[54] Schalm S.S., Blenis J. (2002). Identification of a conserved motif required for mTOR signaling. Curr. Biol.

[55] Dennis P.B., Pullen N., Kozma S.C., Thomas G. (1996). The principal rapamycin-sensitive p70(s6k) phosphorylation sites, T-229 and T-389, are differentially regulated by rapamycin-insensitive kinase kinases. Mol. Cell Biol.

[56] Faivre S., Kroemer G., Raymond E. (2006). Current development of mTOR inhibitors as anticancer agents. Nat. Rev. Drug Discov.

[57] Sonenberg N., Gingras A.C. (1998). The mRNA 5′ cap-binding protein eIF4E and control of cell growth. Curr. Opin. Cell Biol.

[58] Pause A., Belsham G.J., Gingras A.C., Donze O., Lin T.A., Lawrence J.C., Sonenberg N. (1994). Insulin-dependent stimulation of protein synthesis by phosphorylation of a regulator of 5′-cap function. Nature.

[59] Jastrzebski K., Hannan K.M., Tchoubrieva E.B., Hannan R.D., Pearson R.B. (2007). Coordinate regulation of ribosome biogenesis and function by the ribosomal protein S6 kinase, a key mediator of mTOR function. Growth Factors.

[60] Harrington L.S., Findlay G.M., Gray A., Tolkacheva T., Wigfield S., Rebholz H., Barnett J., Leslie N.R., Cheng S., Shepherd P.R. (2004). The TSC1-2 tumor suppressor controls insulin-PI3K signaling via regulation of IRS proteins. J. Cell Biol.

[61] Shah O.J., Wang Z., Hunter T. (2004). Inappropriate activation of the TSC/Rheb/mTOR/S6K cassette induces IRS1/2 depletion, insulin resistance, and cell survival deficiencies. Curr. Biol.

[62] Manning B.D., Cantley L.C. (2003). Rheb fills a GAP between TSC and TOR. Trends Biochem. Sci.

[63] Carracedo A., Ma L., Teruya-Feldstein J., Rojo F., Salmena L., Alimonti A., Egia A., Sasaki A.T., Thomas G., Kozma S.C. (2008). Inhibition of mTORC1 leads to MAPK pathway activation through a PI3K-dependent feedback loop in human cancer. J. Clin. Invest.

[64] Vander Haar E., Lee S.I., Bandhakavi S., Griffin T.J., Kim D.H. (2007). Insulin signalling to mTOR mediated by the Akt/PKB substrate PRAS40. Nat. Cell Biol.

[65] Engelman J.A., Luo J., Cantley L.C. (2006). The evolution of phosphatidylinositol 3-kinases as regulators of growth and metabolism. Nat. Rev. Genet.

[66] Dann S.G., Thomas G. (2006). The amino acid sensitive TOR pathway from yeast to mammals. FEBS Lett.

[67] Kimball S.R., Jefferson L.S. (2006). Signaling pathways and molecular mechanisms through which branched-chain amino acids mediate translational control of protein synthesis. J. Nutr.

[68] Nobukuni T., Joaquin M., Roccio M., Dann S.G., Kim S.Y., Gulati P., Byfield M.P., Backer J.M., Natt F., Bos J.L. (2005). Amino acids mediate mTOR/raptor signaling through activation of class 3 phosphatidylinositol 3OH-kinase. Proc. Natl. Acad. Sci. USA.

[69] Byfield M.P., Murray J.T., Backer J.M. (2005). hVps34 is a nutrient-regulated lipid kinase required for activation of p70 S6 kinase. J. Biol. Chem.

[70] Inoki K., Zhu T., Guan K.L. (2003). TSC2 mediates cellular energy response to control cell growth and survival. Cell.

[71] Hahn-Windgassen A., Nogueira V., Chen C.C., Skeen J.E., Sonenberg N., Hay N. (2005). Akt activates the mammalian target of rapamycin by regulating cellular ATP level and AMPK activity. J. Biol. Chem.

[72] Sofer A., Lei K., Johannessen C.M., Ellisen L.W. (2005). Regulation of mTOR and cell growth in response to energy stress by REDD1. Mol. Cell Biol.

[73] Brugarolas J., Lei K., Hurley R.L., Manning B.D., Reiling J.H., Hafen E., Witters L.A., Ellisen L.W., Kaelin W.G. (2004). Regulation of mTOR function in response to hypoxia by REDD1 and the TSC1/TSC2 tumor suppressor complex. Genes Dev..

[74] Shoshani T., Faerman A., Mett I., Zelin E., Tenne T., Gorodin S., Moshel Y., Elbaz S., Budanov A., Chajut A. (2002). Identification of a novel hypoxia-inducible factor 1-responsive gene, *RTP801*, involved in apoptosis. Mol. Cell Biol.

[75] Liu L., Cash T.P., Jones R.G., Keith B., Thompson C.B., Simon M.C. (2006). Hypoxia-induced energy stress regulates mRNA translation and cell growth. Mol. Cell.

[76] Wolthuis R.M., Bos J.L. (1999). Ras caught in another affair: The exchange factors for Ral. Curr. Opin. Genet. Dev.

[77] Repasky G.A., Chenette E.J., Der C.J. (2004). Renewing the conspiracy theory debate: Does Raf function alone to mediate Ras oncogenesis?. Trends Cell Biol.

[78] Roberts P.J., Der C.J. (2007). Targeting the Raf-MEK-ERK mitogen-activated protein kinase cascade for the treatment of cancer. Oncogene.

[79] Schubbert S., Bollag G., Shannon K. (2007). Deregulated Ras signaling in developmental disorders: New tricks for an old dog. Curr. Opin. Genet. Dev.

[80] Ballif B.A., Roux P.P., Gerber S.A., MacKeigan J.P., Blenis J., Gygi S.P. (2005). Quantitative phosphorylation profiling of the ERK/p90 ribosomal S6 kinase-signaling cassette and its targets, the tuberous sclerosis tumor suppressors. Proc. Natl. Acad. Sci. USA.

[81] Ma L., Chen Z., Erdjument-Bromage H., Tempst P., Pandolfi P.P. (2005). Phosphorylation and functional inactivation of TSC2 by Erk implications for tuberous sclerosis and cancer pathogenesis. Cell.

[82] Ma L., Teruya-Feldstein J., Bonner P., Bernardi R., Franz D.N., Witte D., Cordon-Cardo C., Pandolfi P.P. (2007). Identification of S664 TSC2 phosphorylation as a marker for extracellular signal-regulated kinase mediated mTOR activation in tuberous sclerosis and human cancer. Cancer Res.

[83] Carriere A., Ray H., Blenis J., Roux P.P. (2008). The RSK factors of activating the Ras/MAPK signaling cascade. Front. Biosci.

[84] Pyronnet S., Imataka H., Gingras A.C., Fukunaga R., Hunter T., Sonenberg N. (1999). Human eukaryotic translation initiation factor 4G (eIF4G) recruits mnk1 to phosphorylate eIF4E. EMBO J.

[85] Scheper G.C., Morrice N.A., Kleijn M., Proud C.G. (2001). The mitogen-activated protein kinase signal-integrating kinase Mnk2 is a eukaryotic initiation factor 4E kinase with high levels of basal activity in mammalian cells. Mol. Cell Biol.

[86] Ozes O.N., Akca H., Mayo L.D., Gustin J.A., Maehama T., Dixon J.E., Donner D.B. (2001). A phosphatidylinositol 3-kinase/Akt/mTOR pathway mediates and PTEN antagonizes tumor necrosis factor inhibition of insulin signaling through insulin receptor substrate-1. Proc. Natl. Acad. Sci. USA.

[87] Glantschnig H., Fisher J.E., Wesolowski G., Rodan G.A., Reszka A.A. (2003). M-CSF, TNFα and RANK ligand promote osteoclast survival by signaling through mTOR/S6 kinase. Cell Death Differ.

[88] Lee D.F., Kuo H.P., Chen C.T., Hsu J.M., Chou C.K., Wei Y., Sun H.L., Li L.Y., Ping B., Huang W.C. (2007). IKK β suppression of TSC1 links inflammation and tumor angiogenesis via the mTOR pathway. Cell.

[89] Magnusson C., Vaux D.L. (1999). Signalling by CD95 and TNF receptors: Not only life and death. Immunol. Cell Biol.

[90] Karin M. (2008). The IκB kinase—a bridge between inflammation and cancer. Cell Res.

[91] Dan H.C., Baldwin A.S. (2008). Differential involvement of IκB kinases α and β in cytokine- and insulin-induced mammalian target of rapamycin activation determined by Akt. J. Immunol.

[92] Jacinto E., Facchinetti V., Liu D., Soto N., Wei S., Jung S.Y., Huang Q., Qin J., Su B. (2006). SIN1/MIP1 maintains rictor-mTOR complex integrity and regulates Akt phosphorylation and substrate specificity. Cell.

[93] Huang J., Dibble C.C., Matsuzaki M., Manning B.D. (2008). The TSC1-TSC2 complex is required for proper activation of mTOR complex 2. Mol. Cell Biol.

[94] Tremblay F., Brule S., Hee Um S., Li Y., Masuda K., Roden M., Sun X.J., Krebs M., Polakiewicz R.D., Thomas G., Marette A. (2007). Identification of IRS-1 Ser-1101 as a target of S6K1 in nutrient- and obesity-induced insulin resistance. Proc. Natl. Acad. Sci. USA.

[95] Oldham S., Montagne J., Radimerski T., Thomas G., Hafen E. (2000). Genetic and biochemical characterization of dTOR, the Drosophila homolog of the target of rapamycin. Genes Dev.

[96] Fingar D.C., Salama S., Tsou C., Harlow E., Blenis J. (2002). Mammalian cell size is controlled by mTOR and its downstream targets S6K1 and 4EBP1/eIF4E. Genes Dev.

[97] Audhya A., Loewith R., Parsons A.B., Gao L., Tabuchi M., Zhou H., Boone C., Hall M.N., Emr S.D. (2004). Genome-wide lethality screen identifies new PI4,5P2 effectors that regulate the actin cytoskeleton. EMBO J.

[98] Fadri M., Daquinag A., Wang S., Xue T., Kunz J. (2005). The pleckstrin homology domain proteins Slm1 and Slm2 are required for actin cytoskeleton organization in yeast and bind phosphatidylinositol-4,5-bisphosphate and TORC2. Mol. Biol. Cell.

[99] Peng T., Golub T.R., Sabatini D.M. (2002). The immunosuppressant rapamycin mimics a starvation-like signal distinct from amino acid and glucose deprivation. Mol. Cell Biol.

[100] Hannan K.M., Brandenburger Y., Jenkins A., Sharkey K., Cavanaugh A., Rothblum L., Moss T., Poortinga G., McArthur G.A., Pearson R.B. (2003). mTOR-dependent regulation of ribosomal gene transcription requires S6K1 and is mediated by phosphorylation of the carboxy-terminal activation domain of the nucleolar transcription factor UBF. Mol. Cell Biol.

[101] Mayer C., Zhao J., Yuan X., Grummt I. (2004). mTOR-dependent activation of the transcription factor TIF-IA links rRNA synthesis to nutrient availability. Genes Dev.

[102] Kristof A.S., Marks-Konczalik J., Billings E., Moss J. (2003). Stimulation of signal transducer and activator of transcription-1 (STAT1)-dependent gene transcription by lipopolysaccharide and interferon-gamma is regulated by mammalian target of rapamycin. J. Biol. Chem.

[103] Cardenas M.E., Cutler N.S., Lorenz M.C., Di Como C.J., Heitman J. (1999). The TOR signaling cascade regulates gene expression in response to nutrients. Genes Dev.

[104] Hardwick J.S., Kuruvilla F.G., Tong J.K., Shamji A.F., Schreiber S.L. (1999). Rapamycin-modulated transcription defines the subset of nutrient-sensitive signaling pathways directly controlled by the Tor proteins. Proc. Natl. Acad. Sci. USA.

[105] Powers T., Walter P. (1999). Regulation of ribosome biogenesis by the rapamycin-sensitive TOR-signaling pathway in Saccharomyces cerevisiae. Mol. Biol. Cell.

[106] Levine B., Kroemer G. (2008). Autophagy in the pathogenesis of disease. Cell.

[107] Mizushima N., Levine B., Cuervo A.M., Klionsky D.J. (2008). Autophagy fights disease through cellular self-digestion. Nature.

[108] Levine B., Klionsky D.J. (2004). Development by self-digestion: Molecular mechanisms and biological functions of autophagy. Dev. Cell.

[109] Wu H., Yang J.M., Jin S., Zhang H., Hait W.N. (2006). Elongation factor-2 kinase regulates autophagy in human glioblastoma cells. Cancer Res.

[110] Yu L., McPhee C.K., Zheng L., Mardones G.A., Rong Y., Peng J., Mi N., Zhao Y., Liu Z., Wan F. (2010). Termination of autophagy and reformation of lysosomes regulated by mTOR. Nature.

[111] Tee A.R., Blenis J. (2005). mTOR, translational control and human disease. Semin. Cell Dev. Biol.

[112] Thomas G.V., Horvath S., Smith B.L., Crosby K., Lebel L.A., Schrage M., Said J., de Kernion J., Reiter R.E., Sawyers C.L. (2004). Antibody-based profiling of the phosphoinositide 3-kinase pathway in clinical prostate cancer. Clin. Cancer Res.

[113] Kim J.E., Chen J. (2004). regulation of peroxisome proliferator-activated receptor-gamma activity by mammalian target of rapamycin and amino acids in adipogenesis. Diabetes.

[114] Lazar M.A. (2005). PPAR gamma, 10 years later. Biochimie.

[115] Um S.H., Frigerio F., Watanabe M., Picard F., Joaquin M., Sticker M., Fumagalli S., Allegrini P.R., Kozma S.C., Auwerx J., Thomas G. (2004). Absence of S6K1 protects against age- and diet-induced obesity while enhancing insulin sensitivity. Nature.

[116] Martin D.E., Hall M.N. (2005). The expanding TOR signaling network. Curr. Opin. Cell Biol.

[117] Colman R.J., Anderson R.M., Johnson S.C., Kastman E.K., Kosmatka K.J., Beasley T.M., Allison D.B., Cruzen C., Simmons H.A., Kemnitz J.W. (2009). Caloric restriction delays disease onset and mortality in rhesus monkeys. Science.

[118] Harrison D.E., Strong R., Sharp Z.D., Nelson J.F., Astle C.M., Flurkey K., Nadon N.L., Wilkinson J.E., Frenkel K., Carter C.S. (2009). Rapamycin fed late in life extends lifespan in genetically heterogeneous mice. Nature.

[119] Hanahan D., Weinberg R.A. (2011). Hallmarks of cancer: The next generation. Cell.

[120] Sato T., Nakashima A., Guo L., Coffman K., Tamanoi F. (2010). Single amino-acid changes that confer constitutive activation of mTOR are discovered in human cancer. Oncogene.

[121] Zhou B.P., Hu M.C., Miller S.A., Yu Z., Xia W., Lin S.Y., Hung M.C. (2000). HER-2/neu blocks tumor necrosis factor-induced apoptosis via the Akt/NF-κB pathway. J. Biol. Chem.

[122] Chung J., Bachelder R.E., Lipscomb E.A., Shaw L.M., Mercurio A.M. (2002). Integrin (α6β4) regulation of eIF-4E activity and VEGF translation: A survival mechanism for carcinoma cells. J. Cell Biol.

[123] Stemke-Hale K., Gonzalez-Angulo A.M., Lluch A., Neve R.M., Kuo W.L., Davies M., Carey M., Hu Z., Guan Y., Sahin A. (2008). An integrative genomic and proteomic analysis of PIK3CA, PTEN, and AKT mutations in breast cancer. Cancer Res.

[124] Sansal I., Sellers W.R. (2004). The biology and clinical relevance of the PTEN tumor suppressor pathway. J. Clin. Oncol.

[125] Tamguney T., Stokoe D. (2007). New insights into PTEN. J. Cell Sci.

[126] Vivanco I., Sawyers C.L. (2002). The phosphatidylinositol 3-Kinase AKT pathway in human cancer. Nat. Rev. Cancer.

[127] Armengol G., Rojo F., Castellvi J., Iglesias C., Cuatrecasas M., Pons B., Baselga J., Ramon y Cajal S. (2007). 4E-binding protein 1: A key molecular “funnel factor” in human cancer with clinical implications. Cancer Res.

[128] Coleman L.J., Peter M.B., Teall T.J., Brannan R.A., Hanby A.M., Honarpisheh H., Shaaban A.M., Smith L., Speirs V., Verghese E.T. (2009). Combined analysis of eIF4E and 4E-binding protein expression predicts breast cancer survival and estimates eIF4E activity. Br. J. Cancer.

[129] No J.H., Jeon Y.T., Park I.A., Kim Y.B., Kim J.W., Park N.H., Kang S.B., Han J.Y., Lim J.M., Song Y.S. (2011). Activation of mTOR signaling pathway associated with adverse prognostic factors of epithelial ovarian cancer. Gynecol. Oncol.

[130] Bjornsti M.A., Houghton P.J. (2004). Lost in translation: Dysregulation of cap-dependent translation and cancer. Cancer Cell.

[131] Ruggero D., Montanaro L., Ma L., Xu W., Londei P., Cordon-Cardo C., Pandolfi P.P. (2004). The translation factor eIF-4E promotes tumor formation and cooperates with c-Myc in lymphomagenesis. Nat. Med.

[132] Li J., Yen C., Liaw D., Podsypanina K., Bose S., Wang S.I., Puc J., Miliaresis C., Rodgers L., McCombie R. (1997). *PTEN*, a putative protein tyrosine phosphatase gene mutated in human brain, breast, and prostate cancer. Science.

[133] Campbell I.G., Russell S.E., Choong D.Y., Montgomery K.G., Ciavarella M.L., Hooi C.S., Cristiano B.E., Pearson R.B., Phillips W.A. (2004). Mutation of the *PIK3CA* gene in ovarian and breast cancer. Cancer Res.

[134] Shaw R.J., Cantley L.C. (2006). Ras, PI(3)K and mTOR signalling controls tumour cell growth. Nature.

[135] Basso A.D., Mirza A., Liu G., Long B.J., Bishop W.R., Kirschmeier P. (2005). The farnesyl transferase inhibitor (FTI) SCH66336 (lonafarnib) inhibits Rheb farnesylation and mTOR signaling. Role in FTI enhancement of taxane and tamoxifen anti-tumor activity. J. Biol. Chem.

[136] Lu Z.H., Shvartsman M.B., Lee A.Y., Shao J.M., Murray M.M., Kladney R.D., Fan D., Krajewski S., Chiang G.G., Mills G.B. (2010). Mammalian target of rapamycin activator RHEB is frequently overexpressed in human carcinomas and is critical and sufficient for skin epithelial carcinogenesis. Cancer Res.

[137] Barlund M., Forozan F., Kononen J., Bubendorf L., Chen Y., Bittner M.L., Torhorst J., Haas P., Bucher C., Sauter G. (2000). Detecting activation of ribosomal protein S6 kinase by complementary DNA and tissue microarray analysis. J. Natl. Cancer Inst.

[138] Populo H., Soares P., Faustino A., Rocha A.S., Silva P., Azevedo F., Lopes J.M. (2011). mTOR pathway activation in cutaneous melanoma is associated with poorer prognosis characteristics. Pigment Cell Melanoma Res.

[139] Hiramatsu M., Ninomiya H., Inamura K., Nomura K., Takeuchi K., Satoh Y., Okumura S., Nakagawa K., Yamori T., Matsuura M. (2010). Activation status of receptor tyrosine kinase downstream pathways in primary lung adenocarcinoma with reference of KRAS and EGFR mutations. Lung Cancer.

[140] Noh W.C., Kim Y.H., Kim M.S., Koh J.S., Kim H.A., Moon N.M., Paik N.S. (2008). Activation of the mTOR signaling pathway in breast cancer and its correlation with the clinicopathologic variables. Breast Cancer Res. Treat.

[141] Pantuck A.J., Seligson D.B., Klatte T., Yu H., Leppert J.T., Moore L., O’Toole T., Gibbons J., Belldegrun A.S., Figlin R.A. (2007). Prognostic relevance of the mTOR pathway in renal cell carcinoma: Implications for molecular patient selection for targeted therapy. Cancer.

[142] Zhou L., Huang Y., Li J., Wang Z. (2010). The mTOR pathway is associated with the poor prognosis of human hepatocellular carcinoma. Med. Oncol.

[143] Inoki K., Corradetti M.N., Guan K.L. (2005). Dysregulation of the TSC-mTOR pathway in human disease. Nat. Genet.

[144] Vignot S., Faivre S., Aguirre D., Raymond E. (2005). mTOR-targeted therapy of cancer with rapamycin derivatives. Ann. Oncol.

[145] Hager M., Haufe H., Kemmerling R., Mikuz G., Kolbitsch C., Moser P.L. (2007). PTEN expression in renal cell carcinoma and oncocytoma and prognosis. Pathology.

[146] Madhunapantula S.V., Robertson G.P. (2009). The PTEN-AKT3 signaling cascade as a therapeutic target in melanoma. Pigment Cell Melanoma Res.

[147] Johnson S.R., Tattersfield A.E. (2002). Lymphangioleiomyomatosis. Semin. Respir. Crit. Care Med.

[148] Kwiatkowski D.J. (2003). Tuberous sclerosis: From tubers to mTOR. Ann. Hum. Genet.

[149] Manning B.D., Cantley L.C. (2003). United at last: The tuberous sclerosis complex gene products connect the phosphoinositide 3-kinase/Akt pathway to mammalian target of rapamycin (mTOR) signalling. Biochem. Soc. Trans.

[150] De Benedetti A., Graff J.R. (2004). eIF-4E expression and its role in malignancies and metastases. Oncogene.

[151] Nakamura J.L., Garcia E., Pieper R.O. (2008). S6K1 plays a key role in glial transformation. Cancer Res.

[152] Liaw D., Marsh D.J., Li J., Dahia P.L., Wang S.I., Zheng Z., Bose S., Call K.M., Tsou H.C., Peacocke M. (1997). Germline mutations of the *PTEN* gene in Cowden disease, an inherited breast and thyroid cancer syndrome. Nat. Genet.

[153] Shaw R.J., Bardeesy N., Manning B.D., Lopez L., Kosmatka M., DePinho R.A., Cantley L.C. (2004). The LKB1 tumor suppressor negatively regulates mTOR signaling. Cancer Cell.

[154] Johannessen C.M., Reczek E.E., James M.F., Brems H., Legius E., Cichowski K. (2005). The NF1 tumor suppressor critically regulates TSC2 and mTOR. Proc. Natl. Acad. Sci. USA.

[155] Dahl C., Guldberg P. (2007). The genome and epigenome of malignant melanoma. Apmis.

[156] Dai D.L., Martinka M., Li G. (2005). Prognostic significance of activated Akt expression in melanoma: A clinicopathologic study of 292 cases. J. Clin. Oncol.

[157] Wu H., Goel V., Haluska F.G. (2003). PTEN signaling pathways in melanoma. Oncogene.

[158] Reifenberger J., Wolter M., Bostrom J., Buschges R., Schulte K.W., Megahed M., Ruzicka T., Reifenberger G. (2000). Allelic losses on chromosome arm 10q and mutation of the *PTEN* (*MMAC1*) tumour suppressor gene in primary and metastatic malignant melanomas. Virchows Arch.

[159] Celebi J.T., Shendrik I., Silvers D.N., Peacocke M. (2000). Identification of *PTEN* mutations in metastatic melanoma specimens. J. Med. Genet.

[160] Tsao H., Zhang X., Fowlkes K., Haluska F.G. (2000). Relative reciprocity of NRAS and PTEN/MMAC1 alterations in cutaneous melanoma cell lines. Cancer Res.

[161] Tsao H., Goel V., Wu H., Yang G., Haluska F.G. (2004). Genetic interaction between *NRAS* and *BRAF* mutations and *PTEN*/*MMAC1* inactivation in melanoma. J. Invest. Dermatol.

[162] Davies M.A., Stemke-Hale K., Tellez C., Calderone T.L., Deng W., Prieto V.G., Lazar A.J., Gershenwald J.E., Mills G.B. (2008). A novel *AKT3* mutation in melanoma tumours and cell lines. Br. J. Cancer.

[163] Dhawan P., Singh A.B., Ellis D.L., Richmond A. (2002). Constitutive activation of Akt/protein kinase B in melanoma leads to up-regulation of nuclear factor-κB and tumor progression. Cancer Res.

[164] Stahl J.M., Sharma A., Cheung M., Zimmerman M., Cheng J.Q., Bosenberg M.W., Kester M., Sandirasegarane L., Robertson G.P. (2004). Deregulated Akt3 activity promotes development of malignant melanoma. Cancer Res.

[165] Meier F., Schittek B., Busch S., Garbe C., Smalley K., Satyamoorthy K., Li G., Herlyn M. (2005). The RAS/RAF/MEK/ERK and PI3K/AKT signaling pathways present molecular targets for the effective treatment of advanced melanoma. Front. Biosci.

[166] Cheung M., Sharma A., Madhunapantula S.V., Robertson G.P. (2008). Akt3 and mutant ^V600E^B-Raf cooperate to promote early melanoma development. Cancer Res.

[167] Madhunapantula S.V., Sharma A., Robertson G.P. (2007). PRAS40 deregulates apoptosis in malignant melanoma. Cancer Res.

[168] Saraiva V.S., Caissie A.L., Segal L., Edelstein C., Burnier M.N. (2005). Immunohistochemical expression of phospho-Akt in uveal melanoma. Melanoma Res..

[169] Populo H., Soares P., Rocha A.S., Silva P., Lopes J.M. (2010). Evaluation of the mTOR pathway in ocular (uvea and conjunctiva) melanoma. Melanoma Res.

[170] Abdel-Rahman M.H., Yang Y., Zhou X.P., Craig E.L., Davidorf F.H., Eng C. (2006). High frequency of submicroscopic hemizygous deletion is a major mechanism of loss of expression of PTEN in uveal melanoma. J. Clin. Oncol.

[171] Smalley K.S., Eisen T.G. (2003). Farnesyl transferase inhibitor SCH66336 is cytostatic, pro-apoptotic and enhances chemosensitivity to cisplatin in melanoma cells. Int. J. Cancer.

[172] Easton J.B., Houghton P.J. (2006). mTOR and cancer therapy. Oncogene.

[173] Vezina C., Kudelski A., Sehgal S.N. (1975). Rapamycin (AY-22,989), a new antifungal antibiotic. I. Taxonomy of the producing streptomycete and isolation of the active principle. J. Antibiot. (Tokyo).

[174] Eng C.P., Sehgal S.N., Vezina C. (1984). Activity of rapamycin (AY-22,989) against transplanted tumors. J. Antibiot. (Tokyo).

[175] Linhares M.M., Gonzalez A.M., Trivino T., Melaragno C., Moura R.M., Garcez M.H., Sa J.R., Aguiar W.F., Succi T., Barbosa C.S. (2003). Simultaneous pancreas-kidney transplantation initial experience. Transplant. Proc.

[176] Huang S., Houghton P.J. (2001). Resistance to rapamycin: A novel anticancer drug. Cancer Metastasis Rev.

[177] Dilling M.B., Dias P., Shapiro D.N., Germain G.S., Johnson R.K., Houghton P.J. (1994). Rapamycin selectively inhibits the growth of childhood rhabdomyosarcoma cells through inhibition of signaling via the type I insulin-like growth factor receptor. Cancer Res.

[178] Geoerger B., Kerr K., Tang C.B., Fung K.M., Powell B., Sutton L.N., Phillips P.C., Janss A.J. (2001). Antitumor activity of the rapamycin analog CCI-779 in human primitive neuroectodermal tumor/medulloblastoma models as single agent and in combination chemotherapy. Cancer Res.

[179] Seufferlein T., Rozengurt E. (1996). Rapamycin inhibits constitutive p70s6k phosphorylation, cell proliferation, and colony formation in small cell lung cancer cells. Cancer Res.

[180] Ogawa T., Tokuda M., Tomizawa K., Matsui H., Itano T., Konishi R., Nagahata S., Hatase O. (1998). Osteoblastic differentiation is enhanced by rapamycin in rat osteoblast-like osteosarcoma (ROS 17/2.8) cells. Biochem. Biophys. Res. Commun.

[181] Grewe M., Gansauge F., Schmid R.M., Adler G., Seufferlein T. (1999). Regulation of cell growth and cyclin D1 expression by the constitutively active FRAP-p70s6K pathway in human pancreatic cancer cells. Cancer Res.

[182] Pang H., Faber L.E. (2001). Estrogen and rapamycin effects on cell cycle progression in T47D breast cancer cells. Breast Cancer Res. Treat.

[183] van der Poel H.G., Hanrahan C., Zhong H., Simons J.W. (2003). Rapamycin induces Smad activity in prostate cancer cell lines. Urol. Res.

[184] Muthukkumar S., Ramesh T.M., Bondada S. (1995). Rapamycin, a potent immunosuppressive drug, causes programmed cell death in B lymphoma cells. Transplantation.

[185] Phung T.L., Ziv K., Dabydeen D., Eyiah-Mensah G., Riveros M., Perruzzi C., Sun J., Monahan-Earley R.A., Shiojima I., Nagy J.A. (2006). Pathological angiogenesis is induced by sustained Akt signaling and inhibited by rapamycin. Cancer Cell.

[186] Thomas G.V., Tran C., Mellinghoff I.K., Welsbie D.S., Chan E., Fueger B., Czernin J., Sawyers C.L. (2006). Hypoxia-inducible factor determines sensitivity to inhibitors of mTOR in kidney cancer. Nat. Med.

[187] Gingras A.C., Raught B., Gygi S.P., Niedzwiecka A., Miron M., Burley S.K., Polakiewicz R.D., Wyslouch-Cieszynska A., Aebersold R., Sonenberg N. (2001). Hierarchical phosphorylation of the translation inhibitor 4E-BP1. Genes Dev.

[188] Dumont F.J., Su Q. (1996). Mechanism of action of the immunosuppressant rapamycin. Life Sci.

[189] Dancey J.E. (2006). Therapeutic targets: MTOR and related pathways. Cancer Biol. Ther.

[190] Rini B.I. (2008). Temsirolimus, an inhibitor of mammalian target of rapamycin. Clin. Cancer Res.

[191] Rizzieri D.A., Feldman E., Dipersio J.F., Gabrail N., Stock W., Strair R., Rivera V.M., Albitar M., Bedrosian C.L., Giles F.J. (2008). A phase 2 clinical trial of deforolimus (AP23573, MK-8669), a novel mammalian target of rapamycin inhibitor, in patients with relapsed or refractory hematologic malignancies. Clin. Cancer Res.

[192] Wolpin B.M., Hezel A.F., Abrams T., Blaszkowsky L.S., Meyerhardt J.A., Chan J.A., Enzinger P.C., Allen B., Clark J.W., Ryan D.P. (2009). Oral mTOR inhibitor everolimus in patients with gemcitabine-refractory metastatic pancreatic cancer. J. Clin. Oncol.

[193] Johnston P.B., Inwards D.J., Colgan J.P., Laplant B.R., Kabat B.F., Habermann T.M., Micallef I.N., Porrata L.F., Ansell S.M., Reeder C.B. (2010). A Phase II trial of the oral mTOR inhibitor everolimus in relapsed Hodgkin lymphoma. Am. J. Hematol.

[194] Witzig T.E., Reeder C.B., LaPlant B.R., Gupta M., Johnston P.B., Micallef I.N., Porrata L.F., Ansell S.M., Colgan J.P., Jacobsen E.D. (2011). A phase II trial of the oral mTOR inhibitor everolimus in relapsed aggressive lymphoma. Leukemia.

[195] Ellard S.L., Clemons M., Gelmon K.A., Norris B., Kennecke H., Chia S., Pritchard K., Eisen A., Vandenberg T., Taylor M. (2009). Randomized phase II study comparing two schedules of everolimus in patients with recurrent/metastatic breast cancer: NCIC Clinical Trials Group IND.163. J. Clin. Oncol.

[196] Oza A.M., Elit L., Tsao M.S., Kamel-Reid S., Biagi J., Provencher D.M., Gotlieb W.H., Hoskins P.J., Ghatage P., Tonkin K.S. (2011). Phase II study of temsirolimus in women with recurrent or metastatic endometrial cancer: A trial of the NCIC Clinical Trials Group. J. Clin. Oncol.

[197] Hess G., Herbrecht R., Romaguera J., Verhoef G., Crump M., Gisselbrecht C., Laurell A., Offner F., Strahs A., Berkenblit A. (2009). Phase III study to evaluate temsirolimus compared with investigator’s choice therapy for the treatment of relapsed or refractory mantle cell lymphoma. J. Clin. Oncol.

[198] O’Reilly K.E., Rojo F., She Q.B., Solit D., Mills G.B., Smith D., Lane H., Hofmann F., Hicklin D.J., Ludwig D.L. (2006). mTOR inhibition induces upstream receptor tyrosine kinase signaling and activates Akt. Cancer Res.

[199] Del Bufalo D., Ciuffreda L., Trisciuoglio D., Desideri M., Cognetti F., Zupi G., Milella M. (2006). Antiangiogenic potential of the Mammalian target of rapamycin inhibitor temsirolimus. Cancer Res.

[200] Gossage L., Eisen T. (2010). Alterations in VHL as potential biomarkers in renal-cell carcinoma. Nat. Rev. Clin. Oncol.

[201] Feldman M.E., Apsel B., Uotila A., Loewith R., Knight Z.A., Ruggero D., Shokat K.M. (2009). Active-site inhibitors of mTOR target rapamycin-resistant outputs of mTORC1 and mTORC2. PLoS Biol.

[202] Thoreen C.C., Kang S.A., Chang J.W., Liu Q., Zhang J., Gao Y., Reichling L.J., Sim T., Sabatini D.M., Gray N.S. (2009). An ATP-competitive mammalian target of rapamycin inhibitor reveals rapamycin-resistant functions of mTORC1. J. Biol. Chem.

[203] Molckovsky A., Siu L.L. (2008). First-in-class, first-in-human phase I results of targeted agents: Highlights of the 2008 American society of clinical oncology meeting. J. Hematol. Oncol.

[204] Yap T.A., Garrett M.D., Walton M.I., Raynaud F., de Bono J.S., Workman P. (2008). Targeting the PI3K-AKT-mTOR pathway: Progress, pitfalls, and promises. Curr. Opin. Pharmacol.

[205] Liu T.J., Koul D., LaFortune T., Tiao N., Shen R.J., Maira S.M., Garcia-Echevrria C., Yung W.K. (2009). NVP-BEZ235, a novel dual phosphatidylinositol 3-kinase/mammalian target of rapamycin inhibitor, elicits multifaceted antitumor activities in human gliomas. Mol. Cancer Ther.

[206] Ballou L.M., Lin R.Z. (2008). Rapamycin and mTOR kinase inhibitors. J. Chem. Biol.

[207] Chresta C.M., Davies B.R., Hickson I., Harding T., Cosulich S., Critchlow S.E., Vincent J.P., Ellston R., Jones D., Sini P. (2010). AZD8055 is a potent, selective, and orally bioavailable ATP-competitive mammalian target of rapamycin kinase inhibitor with *in vitro* and *in vivo* antitumor activity. Cancer Res.

[208] Garcia-Martinez J.M., Moran J., Clarke R.G., Gray A., Cosulich S.C., Chresta C.M., Alessi D.R. (2009). Ku-0063794 is a specific inhibitor of the mammalian target of rapamycin (mTOR). Biochem. J.

[209] Yu K., Toral-Barza L., Shi C., Zhang W.G., Lucas J., Shor B., Kim J., Verheijen J., Curran K., Malwitz D.J. (2009). Biochemical, cellular, and *in vivo* activity of novel ATP-competitive and selective inhibitors of the mammalian target of rapamycin. Cancer Res.

[210] Raynaud F.I., Eccles S., Clarke P.A., Hayes A., Nutley B., Alix S., Henley A., Di-Stefano F., Ahmad Z., Guillard S. (2007). Pharmacologic characterization of a potent inhibitor of class I phosphatidylinositide 3-kinases. Cancer Res.

[211] Venkatesan A.M., Chen Z., dos Santos O., Dehnhardt C., Santos E.D., Ayral-Kaloustian S., Mallon R., Hollander I., Feldberg L., Lucas J. (2010). PKI-179: An orally efficacious dual phosphatidylinositol-3-kinase (PI3K)/mammalian target of rapamycin (mTOR) inhibitor. Bioorg. Med. Chem. Lett.

[212] Venkatesan A.M., Dehnhardt C.M., Delos Santos E., Chen Z., Dos Santos O., Ayral-Kaloustian S., Khafizova G., Brooijmans N., Mallon R., Hollander I. (2010). Bis(morpholino-1,3,5-triazine) derivatives: Potent adenosine 5′-triphosphate competitive phosphatidylinositol-3-kinase/mammalian target of rapamycin inhibitors: Discovery of compound 26 (PKI-587), a highly efficacious dual inhibitor. J. Med. Chem.

[213] Grunwald V., DeGraffenried L., Russel D., Friedrichs W.E., Ray R.B., Hidalgo M. (2002). Inhibitors of mTOR reverse doxorubicin resistance conferred by PTEN status in prostate cancer cells. Cancer Res.

[214] Mondesire W.H., Jian W., Zhang H., Ensor J., Hung M.C., Mills G.B., Meric-Bernstam F. (2004). Targeting mammalian target of rapamycin synergistically enhances chemotherapy-induced cytotoxicity in breast cancer cells. Clin. Cancer Res.

[215] Steelman L.S., Navolanic P.M., Sokolosky M.L., Taylor J.R., Lehmann B.D., Chappell W.H., Abrams S.L., Wong E.W., Stadelman K.M., Terrian D.M. (2008). Suppression of PTEN function increases breast cancer chemotherapeutic drug resistance while conferring sensitivity to mTOR inhibitors. Oncogene.

[216] Kreisl T.N., Lassman A.B., Mischel P.S., Rosen N., Scher H.I., Teruya-Feldstein J., Shaffer D., Lis E., Abrey L.E. (2009). A pilot study of everolimus and gefitinib in the treatment of recurrent glioblastoma (GBM). J. Neurooncol.

[217] Reardon D.A., Desjardins A., Vredenburgh J.J., Gururangan S., Friedman A.H., Herndon J.E., Marcello J., Norfleet J.A., McLendon R.E., Sampson J.H. (2010). Phase 2 trial of erlotinib plus sirolimus in adults with recurrent glioblastoma. J. Neurooncol.

[218] Johnston S.R. (2010). New strategies in estrogen receptor-positive breast cancer. Clin. Cancer Res.

[219] Morrow P.K., Wulf G.M., Ensor J., Booser D.J., Moore J.A., Flores P.R., Xiong Y., Zhang S., Krop I.E., Winer E.P. (2011). Phase I/II study of trastuzumab in combination with everolimus (RAD001) in patients with HER2-overexpressing metastatic breast cancer who progressed on trastuzumab-based therapy. J. Clin. Oncol.

[220] Merchan J.R., Pitot H.C., Qin R., Liu G., Fitch T.R., Picus J., Maples W.J., Erlichman C (2009). Phase I/II trial of CCI 779 and bevacizumab in advanced renal cell carcinoma (RCC): Safety and activity in RTKI refractory RCC patients. J. Clin. Oncol.

[221] Patnaik A., Ricart A., Cooper J., Papadopoulos K., Beeram M., Mita C., Mita M.M., Hufnagel D., Izbicka E., Tolcher A.W. (2007). A phase I, pharmacokinetic and pharmacodynamic study of sorafenib (S), a multi-targeted kinase inhibitor in combination with temsirolimus (T), an mTOR inhibitor in patients with advanced solid malignancies. J. Clin. Oncol.

[222] Patel P.H., Senico P.L., Curiel R.E., Motzer R.J. (2009). Phase I study combining treatment with temsirolimus and sunitinib malate in patients with advanced renal cell carcinoma. Clin. Genitourin. Cancer.

[223] Houghton P (2008). Targeting the IGF-1/mTOR pathway.

[224] Kurmasheva R.T., Easton J.B., Houghton P.J. (2008). Combined targeting of mTOR and the insulin-like growth factor pathway. ASCO Educ. Book 2008.

[225] Lasithiotakis K.G., Sinnberg T.W., Schittek B., Flaherty K.T., Kulms D., Maczey E., Garbe C., Meier F.E. (2008). Combined inhibition of MAPK and mTOR signaling inhibits growth, induces cell death, and abrogates invasive growth of melanoma cells. J. Invest. Dermatol.

[226] McDaid H.M., Legrier M., Yang C.H., Yan H.G., Lopez-Barcons L., Keller S.M., Horwitz S.B. (2007). Combined MEK and mTOR suppression is synergistic in human NSCLC and is mediated via inhibition of protein translation. J. Clin. Oncol.

[227] Molhoek K.R., Brautigan D.L., Slingluff C.L. (2005). Synergistic inhibition of human melanoma proliferation by combination treatment with B-Raf inhibitor BAY43-9006 and mTOR inhibitor Rapamycin. J. Transl. Med.

[228] Romano M.F., Avellino R., Petrella A., Bisogni R., Romano S., Venuta S. (2004). Rapamycin inhibits doxorubicin-induced NF-κB/Rel nuclear activity and enhances the apoptosis of melanoma cells. Eur. J. Cancer.

[229] Werzowa J., Cejka D., Fuereder T., Dekrout B., Thallinger C., Pehamberger H., Wacheck V., Pratscher B. (2009). Suppression of mTOR complex 2-dependent AKT phosphorylation in melanoma cells by combined treatment with rapamycin and LY294002. Br. J. Dermatol.

[230] Thallinger C., Poeppl W., Pratscher B., Mayerhofer M., Valent P., Tappeiner G., Joukhadar C. (2007). CCI-779 plus cisplatin is highly effective against human melanoma in a SCID mouse xenotranplantation model. Pharmacology.

[231] Eberle J., Kurbanov B.M., Hossini A.M., Trefzer U., Fecker L.F. (2007). Overcoming apoptosis deficiency of melanoma-hope for new therapeutic approaches. Drug Resist. Updat.

[232] Noh W.C., Mondesire W.H., Peng J., Jian W., Zhang H., Dong J., Mills G.B., Hung M.C., Meric-Bernstam F. (2004). Determinants of rapamycin sensitivity in breast cancer cells. Clin. Cancer Res.

[233] Neshat M.S., Mellinghoff I.K., Tran C., Stiles B., Thomas G., Petersen R., Frost P., Gibbons J.J., Wu H., Sawyers C.L. (2001). Enhanced sensitivity of PTEN-deficient tumors to inhibition of FRAP/mTOR. Proc. Natl. Acad. Sci. USA.

[234] Yu K., Toral-Barza L., Discafani C., Zhang W.G., Skotnicki J., Frost P., Gibbons J.J. (2001). mTOR, a novel target in breast cancer: The effect of CCI-779, an mTOR inhibitor, in preclinical models of breast cancer. Endocr. Relat. Cancer.

[235] Cloughesy T.F., Yoshimoto K., Nghiemphu P., Brown K., Dang J., Zhu S., Hsueh T., Chen Y., Wang W., Youngkin D. (2008). Antitumor activity of rapamycin in a Phase I trial for patients with recurrent PTEN-deficient glioblastoma. PLoS Med.

